# Exploring stakeholder support for student-run free clinics to address gaps in non-communicable disease care in Ghana: An exploratory sequential mixed-methods study

**DOI:** 10.1371/journal.pgph.0005092

**Published:** 2026-09-21

**Authors:** Brian Amu Fleischer, Esi Berkoh, Bismark Amoh, Afriyie Badu, Derek Anamaale Tuoyire, Jeremy I. Schwartz

**Affiliations:** 1 Yale School of Medicine, New Haven, Connecticut, United States of America; 2 Department of Community Medicine, University of Cape Coast School of Medical Sciences, Cape Coast, Ghana; 3 David Geffen School of Medicine at UCLA, University of California, Los Angeles, California, United States of America; 4 Ga East Municipal Hospital, Accra, Ghana; 5 Section of General Internal Medicine, Yale School of Medicine, New Haven, Connecticut, United States of America; PLOS: Public Library of Science, UNITED STATES OF AMERICA

## Abstract

Non-communicable diseases (NCDs), including hypertension and diabetes, are leading causes of morbidity and mortality in Ghana, where underserved communities face persistent barriers to screening, early management, and self-care education. Student-run free clinics (SRFCs) may help address these gaps, but their feasibility in sub-Saharan Africa remains underexplored. We used an exploratory sequential mixed-methods design to examine stakeholder perspectives on and support for SRFCs as a model for expanding NCD care in Ghana. Qualitative data were collected through six focus group discussions with 48 health professional students, six key informant interviews with university faculty, academic leaders, and Ghana Health Service officials, and two focus group discussions with 12 community members. Quantitative surveys of 316 health professional students assessed willingness to participate in SRFCs and associated factors. Thematic analysis, descriptive statistics, and logistic regression were used, and qualitative and quantitative findings were integrated narratively. Stakeholders across all groups expressed strong support for student-led initiatives addressing gaps in NCD care. Findings highlighted students’ familiarity with NCDs and barriers to care; strong acceptance of SRFCs as relevant, feasible, and potentially impactful; and endorsement from institutional and community stakeholders. Participants emphasized community impact, hands-on training, and interprofessional collaboration as potential benefits, while identifying academic demands, resource constraints, and safety concerns as possible barriers. Overall, 86.7% of surveyed students were willing to participate in SRFCs. Greater familiarity with NCDs predicted willingness to participate (odds ratio = 1.41, p = 0.007), while final-year students demonstrated lower willingness than pre-clinical and non-final-year clinical students (p = 0.0096). SRFCs represent a promising and contextually relevant model for complementing existing NCD services in Ghana. Successful implementation will require structured supervision, adequate resources, and sustainable integration with universities, communities, and the national health system.

## Introduction

Student-run free clinics (SFRCs) have emerged as crucial safety nets delivering essential primary medical care to underserved populations [1,2]. Staffed by dedicated students of medicine, nursing, pharmacy, and other health professional programs (herein referred to as “health professional students”) under the supervision of faculty, these clinics help address healthcare disparities and improve public health. In South Africa, the Students’ Health and Welfare Centers Organization (SHAWCO) operates over 200 clinics with more than 1,250 student volunteers across eight provinces [2]. In the United States, over 140,000 patients are seen annually at 208 SRFC sites, with at least 75% of accredited medical schools supporting them [3].

In Ghana, non-communicable diseases (NCDs) pose a significant public health challenge. Hypertension, affecting 34% of Ghanaians, is the leading cause of death and the third leading cause of hospital admissions, accounting for 15.3% of total deaths and 4.7% of hospitalizations [4,5]. Despite this high disease burden, Ghana faces a severe shortage of healthcare providers, with fewer than one physician per 10,000 people [6]. This imbalance between healthcare needs and available resources underscores the urgency for innovative and sustainable interventions. Student-led initiatives, particularly within SRFCs, offer a promising approach to addressing these gaps by extending healthcare access and providing early screening, management, and education for NCDs.

Integrating cost-effective NCD interventions in primary care has been shown to be effective in reversing disease progression, preventing complications, reducing hospitalizations, lowering healthcare costs [7]. Task-shifting, which delegates specific medical responsibilities from doctors to trained non-physician healthcare workers (NPHWs) and community health officers (CHOs), has proven effective in areas with physician shortages [8]. Ghana’s Community-based Health Planning and Services (CHPS) program, originally designed for maternal and child health, exemplifies this approach [8]. Despite the program’s success in transforming maternal and child health over the past 20 years, it lacks the training, medications, and resources necessary to address NCDs. As such, it remains poorly equipped to respond to the NCD epidemic, thereby justifying innovative solutions that build on the current model and other cost-effective interventions [9].

Interprofessional SRFCs has proven to be instrumental in delivering comprehensive care to underserved communities [10]. They create an environment of collaboration, continuous learning, and skill development [11]. Ghana’s health education system offers a rich pool of expertise across medical, pharmacy, nursing, public health, and allied health disciplines [12]. Medical students bring diagnostic and clinical expertise for NCD screening and management. Nursing students offer patient care and counseling to support self-management. Pharmacy students ensure proper medication management and adherence. Public health students contribute community health promotion and disease prevention strategies. Other allied health students, including those in social work, nutrition, and physiotherapy, provide specialized expertise to enhance NCD care. By fostering collaboration among these disciplines, SRFCs could meet unmet needs in NCD care, providing holistic and innovative solutions for underserved communities.

Leveraging the SRFC model in Ghana presents a promising approach to addressing the country’s NCD crisis and, if successful, could serve as a model for the region. This study assesses stakeholder perspectives on the feasibility and support for implementing SRFCs in Ghana. A list of abbreviations used throughout the manuscript is provided in S1 Appendix.

## Methods

### Ethics statement

Ethical approval was obtained from the GHS (GHS-ERC:040/11/23) and Yale University (2000036204) IRBs. Verbal and written consent was obtained from all participants. Before consent forms were signed, participants were provided with an information sheet that was read and explained to them. Participants were informed that participation was entirely voluntary and that they could withdraw at any time without penalty. They were assured that their decision not to participate or to withdraw would not affect their treatment or participation in any other aspect of the study. All data were de-identified and securely stored. No compensation was provided, but refreshments were offered. Data ownership and potential conflicts of interest were disclosed. There were no variations from the study protocol after approval was obtained.

### Study design and setting

This study employed a mixed-methods exploratory sequential design to assess stakeholder interest in health professional student-led interventions for addressing access gaps in hypertension and diabetes management in Ghana [13]. The research was conducted across two major public universities affiliated to teaching hospitals, Ghana Health Service (GHS) institutions, and nearby communities. The study unfolded in two phases: a qualitative phase involving focus group discussions (FGDs) and key informant interviews (KIIs), followed by a quantitative phase featuring a structured survey. Insights from the qualitative phase informed the development of the survey instrument to examined students’ motivation, perceived impact, and feasibility of the SRFC model.

Student and faculty interviews were conducted at the University of Ghana Medical School and the University of Cape Coast School of Medical Sciences. The University of Ghana, located in Accra, is the nation’s largest university [14]. It is affiliated with Korle Bu Teaching Hospital which serves 400,000 patients annually, primarily from Accra’s urban, peri-urban, and slum populations, but also across Ghana and West Africa [15]. The University of Cape Coast, in a less urban city town, is affiliated with Cape Coast Teaching Hospital, a 400-bed facility serving the city and surrounding rural communities [16].

Interviews with GHS community nurses and district health directors were conducted at the Mfantseman Ghana Health Service District Directorate in Saltpond. Interviews with higher-level officials and policymakers were conducted at the Ministry of Health and the Office of the National Presidential Advisor on Health, both located in Accra. Community members FGDs were conducted at Oguaa Market in Cape Coast and Korle Gonno Townsquare in Accra.

### Data collection procedures

#### Qualitative techniques.

Between February 8th, 2024, and April 19^th^, 2024, we conducted FGDs and KIIs to explore stakeholders’ perspectives on non-communicable diseases (NCDs), access barriers, and interest in student-led care models.

- Sampling Strategy: Purposive sampling was used to recruit participants with diverse yet complementary perspectives on non-communicable disease (NCD) care and medical education. Health professional students enrolled in the participating universities were included because they represent the future clinical workforce and are central to the feasibility of a student-run clinic model. Participants were drawn from different levels and disciplines—Medicine, Nursing, Pharmacy, Optometry, and Allied Health Sciences to capture variation in clinical exposure, interprofessional learning, and teamwork experience. Key informants were selected based on their decision-making authority and direct involvement in community-based training, curriculum design, or NCD program implementation—specifically, university deans, faculty supervisors, and Ghana Health Service officials at district and national levels.

Community members were adult residents (≥18 years) from underserved communities within the catchment areas of affiliated teaching hospitals and nearby markets or towns. Participants were selected because they represented potential beneficiaries of community-based NCD screening, education, referral, and early management services. While some participants reported a personal history of NCDs, inclusion was not restricted to individuals living with chronic disease, as broader community perspectives were considered important for understanding healthcare access barriers and acceptability of student-led interventions. This combination of stakeholders was designed to ensure representation from the educational, policy, and community perspectives most relevant to assessing the feasibility and contextual appropriateness of student-run free clinics (SRFCs) in Ghana. Exclusion criteria included minors (<18 years), pregnant individuals, and persons unable to provide informed consent or participate meaningfully due to cognitive or language limitations.

- Data Saturation: Qualitative data were collected through six focus group discussions (FGDs) with 48 health professional students, six key informant interviews (KIIs) with faculty, deans, and Ghana Health Service (GHS) officials, and two FGDs with 12 community members. Data collection continued until thematic saturation was reached—defined as the point at which no new codes or substantive ideas emerged from consecutive interviews or focus groups. Saturation was assessed iteratively by the research team as transcripts were reviewed and coded. By the final student FGDs and KIIs, the major code categories had stabilized, with only minor refinements to subthemes in the community discussions. Two additional sessions were conducted thereafter to confirm consistency and ensure adequate representation across stakeholder groups.
- Data Collection Summary:
  - 6 FGDs with 48 health professional students
  - 6 KIIs with university faculty (deans, lecturers)
  - 4 KIIs with GHS officials, including public health nurses, district directors and national level policymakers.
  - 2 FGDs with 12 community members in Cape Coast and Accra
- Procedures: All discussions used a semi-structured guide covering NCD knowledge, perceived access gaps, and attitudes toward student-run interventions. Interviews and FGDs were conducted in English or local languages (Twi/Fante), audio-recorded with permission, and transcribed verbatim. Translations were reviewed by bilingual team members for accuracy. Prior to discussing Student-Run Free Clinics (SRFCs), moderators provided participants with a standardized overview of the SRFC model. This overview included the purpose and structure of SRFCs, examples of established programs in both the United States and Africa—including HAVEN Free Clinic at Yale University, SHAWCO at the University of Cape Town, and other student-run clinics operating in academic medical centers across the United States—the expected roles and responsibilities of health professional students, limits of student practice, faculty supervision requirements, and mechanisms for regulatory oversight and patient safety. Participants were given the opportunity to ask questions and seek clarification before proceeding with the discussion to ensure a shared understanding of the concept. We conducted a reflexive thematic analysis following Braun and Clarke’s six-phase framework, moving from inductive open coding to interpretive theme development focused on explanatory patterns (e.g., how students balance academic demands with community service motivation) rather than purely descriptive grouping. Themes were refined through iterative discussion among analysts to identify underlying relationships and contextual meanings relevant to SRFC feasibility.

#### Quantitative techniques.

The quantitative phase involved a structured survey administered from April 24^th^ to April 30^th^, 2024, to assess students’ attitudes toward participating in student-run clinics and perceptions of NCD care.

- Sampling and Administration: The survey was distributed to 316 health professional students across both universities using an online questionnaire. Students were recruited via academic listservs, in-class announcements, and student organization outreach. Participation was voluntary and anonymous, with electronic consent obtained beforehand. Prior to answering survey questions related to student-run free clinics (SRFCs), respondents were provided with a brief standardized description of the SRFC model, including its focus on non-communicable disease screening, early management, and health education, as well as the role of health professional students working under faculty supervision. This description was included to ensure a common understanding of the intervention being assessed.
- Instrument Design: The survey included items derived from qualitative themes, structured around Likert-scale and multiple-choice questions assessing:
  - Self-reported familiarity with NCDs using a 5-point Likert scale.
  - Familiarity with common NCDs through identification of major NCD conditions.
  - Perceived burden of common NCDs in Ghana.
  - Perceived barriers to NCD screening, early management and self-care education.
  - Interest in leading or participating in SRFCs.

The self-reported familiarity measure was intended to capture students’ overall perceived awareness and exposure to NCDs rather than a specific domain of knowledge. This item was interpreted alongside responses regarding common NCDs and barriers to NCD care to provide a broader understanding of students’ familiarity with NCD-related health challenges. Operational definitions and coding procedures for the quantitative variables are provided in S2 Table.

### Data analysis

#### Qualitative phase.

Thematic analysis was performed using NVivo (Release 1.7.2, QSR International, 2022) [17] to identify recurring patterns and themes. Transcripts were first prepared and thoroughly reviewed by four researchers (B.F., E.B., A.B., and B.A.) to ensure familiarization with the data.

Two analysts (B.A.F and B.A) independently coded an initial subset of transcripts to establish a shared understanding of code definitions and boundaries. Coding discrepancies were reviewed through joint discussion until full consensus was reached, and the refined codebook was then applied to the remaining transcripts. To maintain consistency, approximately 20% of all transcripts were double-coded and reviewed together during analysis. While a formal statistic such as Cohen’s κ was not calculated, analytic reliability was ensured through iterative consensus meetings, codebook audits, and transparent documentation of decision changes. Member-checking was conducted with a subset of participants (students = 6, faculty/GHS = 3, community = 4) who reviewed a one-page summary of preliminary findings. Their feedback largely affirmed the thematic structure and provided additional emphasis on practical barriers, such as transport cost and safety, which were integrated into the final analysis. The qualitative coding and validation procedures are summarized in S1 Table.

#### Quantitative phase.

**Instrument development and validation:** The quantitative questionnaire was developed directly from the themes identified in the qualitative phase. Findings from the focus group discussions — including perceptions of NCD burden, access barriers, enabling and limiting factors for student-led interventions, and motivational drivers — informed the structure and content of the survey. Corresponding items were grouped into domains reflecting (1) awareness and interest in student-led interventions, (2) motivations and factors influencing participation, (3) preferred intervention types, (4) perceived facilitators for implementation, and (5) overall confidence and willingness to participate.

To establish content and face validity**,** the draft instrument was reviewed by two faculty mentors and two Ghana Health Service officials with experience in NCD programming and community-based education. Their feedback helped refine item phrasing, eliminate redundancy, and ensure contextual relevance to the Ghanaian primary care setting. The revised survey was pilot tested among 10 health professional students to evaluate clarity, flow, and response time. Based on feedback, minor modifications were made (e.g., simplifying technical language and balancing Likert-scale response options). Given the categorical and exploratory nature of the items, formal reliability statistics such as Cronbach’s α were not calculated; however, item coherence was confirmed through internal review of response consistency and expert agreement on construct alignment. The final questionnaire included 12 items covering demographics, prior exposure to student-led interventions, perceived barriers and enablers, and willingness to participate.

**Sample size justification:** We planned the survey to estimate key proportions (e.g., willingness to participate in SRFCs) with adequate precision. Using the single-proportion formula


$$\begin{array}{c} n=\frac{{Z_{\text{0}.\text{9}\text{5}}}^{\text{2}}p(\text{1}-p)}{d^{\text{2}}}, \end{array}$$

with a conservative expected proportion p = 0.50 (maximizes required n), 95% confidence (Z = 1.96) and absolute precision d = 0.05, the target sample size was n = 384. Data collection yielded **316** completed surveys within the study window. This achieved sample provides a 95% margin of error of approximately **±5.5%** at worst case (computed as 1.960$\sqrt{\text{0}.\text{2}\text{5}/\text{3}\text{1}\text{6}}$).

For the multivariable analysis, events-per-variable (EPV) criteria were satisfied. With 274 “willing to participate” responses, EPV far exceeded the conventional threshold of 10 for our primary model (one key predictor), supporting stable logistic regression estimates.

Survey data were analyzed using SPSS version 28 [18]

- Descriptive Statistics: Used to summarize participant demographics, NCD familiarity, and levels of interest in SRFC involvement.
- Bivariate Analysis: Associations between categorical variables were examined using Chi-square (χ²) tests. The assumptions of independence of observations and expected cell frequencies (≥ 5 in at least 80% of cells) were verified. Where these assumptions were violated due to small subgroup counts, Fisher-Freeman-Halton Exact Test was applied. Statistical significance was set at *p* < 0.05 for all analyses.
- Multivariable Analysis: Logistic regression examined whether familiarity with NCDs predicted willingness to lead or participate in SRFCs.
- Statistical Threshold: A p-value of <0.05 was considered statistically significant.

## Results

### Health professional participant demographics

Health professional students’ demographic characteristics for the qualitative and quantitative phases are summarized in Table 1 and Table 2, respectively. A total of 48 students participated in the qualitative focus group discussions, while 316 students completed the quantitative survey. The mean age across both cohorts was approximately 22 years, with the qualitative sample ranging from 17 to 27 years (SD = 2.49) and the survey sample from 17 to 36 years (SD = 3.11). Gender distribution was relatively balanced, with females comprising 45.8% of qualitative participants and 52.8% of survey respondents. Participants represented a range of health professional programs—including nursing, pharmacy, allied health sciences, medicine, and optometry—with nursing and pharmacy students being the most represented in the survey phase (25.9% and 25.6%, respectively). Students were distributed across all academic levels, from first year (Level 100) to final year (Level 600), providing a broad spectrum of perspectives across the educational continuum.

**Table 1:** Demographic characteristics of health-professional students participating in the qualitative and quantitative phases. Summary of participant demographics for focus group discussion (FGD) participants and survey respondents. Data are presented as frequencies (n [%]) for categorical variables and mean (SD) with range for continuous variables.

Characteristic	FGD Participants (N = 48)	Survey Respondents (N = 316)
**Age (years)**		
Mean (SD)	22.4 (2.49)	22.3 (3.11)
Range	17 – 27	17 – 36
**Gender, n (%)**		
Female	22 (45.8)	167 (52.8)
Male	26 (52.2)	149 (47.2)
**Health Professional Program, n (%)**		
Nursing	12 (25.0)	82 (25.9)
Pharmacy	8 (16.7)	81 (25.6)
Allied Health Sciences	12 (25.0)	72 (22.8)
Medicine	10 (20.8)	55 (17.4)
Optometry	6 (12.5)	17 (5.3)
Other	—	9 (2.8)
**Academic level, n (%)**		
Level 100	7 (14.6)	73 (23.1)
Level 200	12 (25.0)	65 (20.1)
Level 300	11 (22.9)	39 (12.3)
Level 400	6 (12.5)	64 (20.3)
Level 500	8 (16.7)	62 (19.6)
Level 600	4 (8.3)	13 (4.1)

**Table 2:** Bivariate associations between academic characteristics and willingness to participate in student-run free clinics (SRFCs) among health professional students. Fisher-Freeman-Halton Exact Test was used for all comparisons.

Academic characteristics vs. willingness to participate				
Variable	Category	Yes, n (%)	No, n (%)	p-value
**Academic Program**	Allied Health Sciences	60 (83.3)	12 (16.7)	0.0686
	Nursing	65 (79.3)	17 (20.7)	
	Pharmacy	75 (92.6)	6 (7.4)	
	Medicine	51 (92.7)	4 (7.3)	
	Optometry	16 (94.1)	1 (5.9)	
	Other	7 (77.8)	2 (22.2)	
**Training Level**	Pre-clinical	93 (88.6)	12 (11.4)	0.0096
	Clinical (non-final year)	132 (90.4)	14 (9.6)	
	Final Year	49 (75.4)	16 (24.6)	

### Stakeholder perspectives on SRFCs: integrated findings

Findings from both the qualitative and quantitative phases were integrated at the interpretation level using a narrative weaving approach. This mixed-methods integration allowed for a richer understanding of stakeholder perspectives on student-led responses to the growing burden of non-communicable diseases (NCDs) in Ghana. Across health professional students, university faculty, Ghana Health Service (GHS) officials, and community leaders, there was strong and consistent support for the concept of a Student-Run Free Clinic (SRFC) focused on NCD screening, early management, and self-care education.

Importantly, this support was shaped by both lived experiences and structural realities. Students not only demonstrated a deep familiarity with the landscape of NCDs in Ghana but also articulated the complex barriers that impede care—from sociocultural attitudes and low health literacy to systemic constraints like cost and infrastructure gaps. Their belief in the relevance of SRFCs was tied directly to how well the model aligned with these observed needs. At the same time, stakeholders across sectors recognized SRFCs as a feasible and complementary intervention—one that could extend the reach of existing health systems, support national priorities, and provide meaningful training opportunities for future health professionals.

To better understand the depth and contours of this support, three key domains emerged from our analysis: (1) students’ high level of familiarity with NCDs and perceived barriers to care; (2) strong acceptance of SRFCs as a viable and contextually appropriate intervention among students; and (3) widespread cross-sector endorsement from GHS, university leadership, and community stakeholders. These domains are explored in detail below.

### Key domain 1: high level of familiarity with NCDs and barriers to care among students

Health professional students demonstrated a high level of familiarity with non-communicable diseases (NCDs), shaped by personal, academic, and clinical exposure. During focus group discussions, many students shared stories about family members, peers, or acquaintances affected by NCDs. They cited conditions such as hypertension, diabetes, cancer, and stroke, and described basic mechanisms of disease, long-term complications, and the broader health system impact. These reflections indicated both theoretical knowledge and lived experience.

“My father had liver cancer but before that, he had diabetes, a terminal one. So, he passed away last year.” (University of Cape Coast Group 2 Student 2, UC2S2).

“My roommate was living with diabetes. It’s not a good experience; trust me”. (UC1S5).

“When I hear non-communicable diseases, what usually comes to mind is conditions like diabetes, hypertension, [] because they can’t be transmitted from one person to the other.” (University of Ghana Group 2 Student 3, UG2S3).

**Fig 1:**
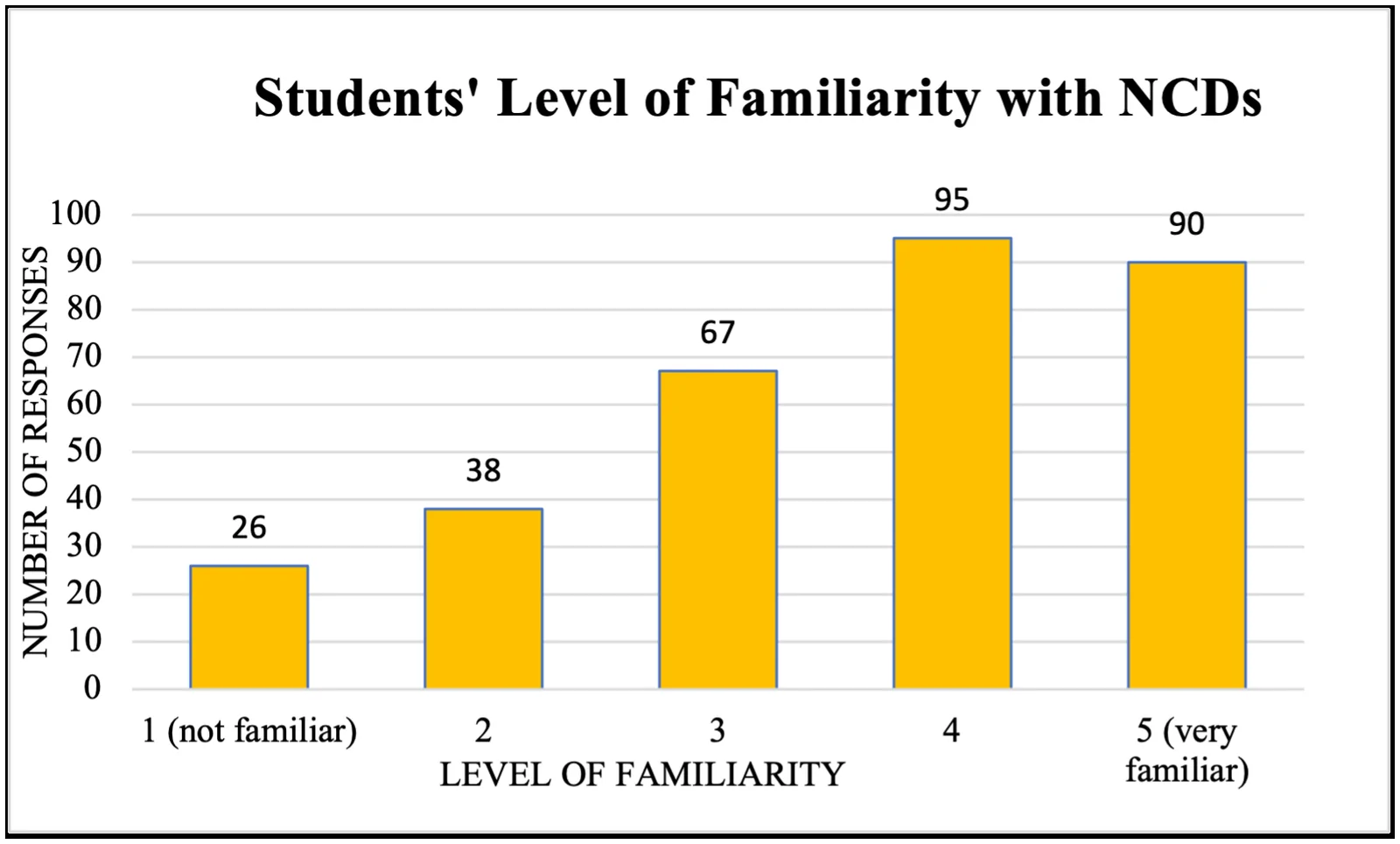
Health professional students’ rating of their familiarity with NCDs (1 = No familiarity; 5 = High familiarity).

**Fig 2:**
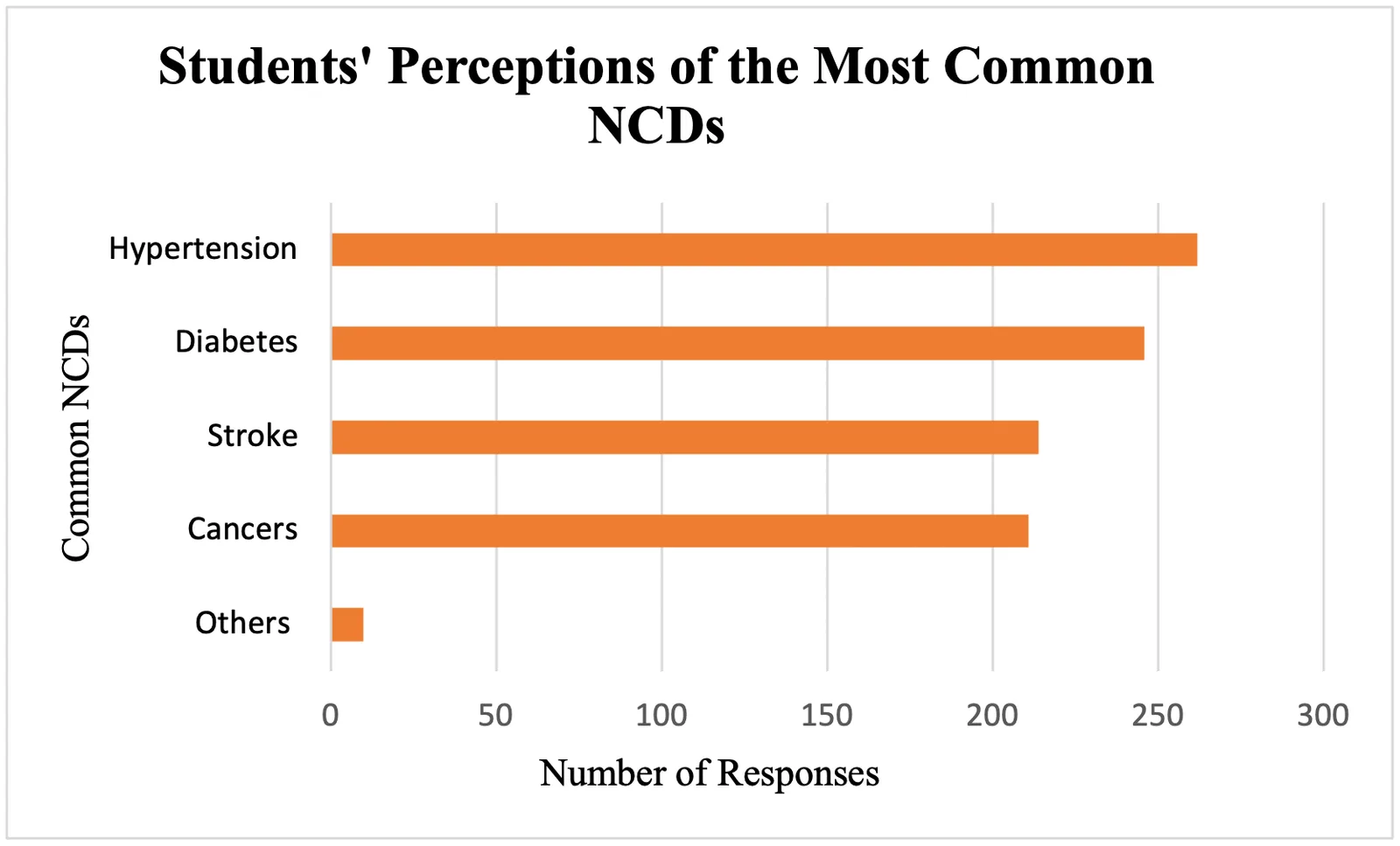
Health professional students’ perception of the most common NCDs. “Others” include asthma, sickle cell anemia, jaundice, and thyrotoxicosis.

These qualitative findings were mirrored in the structured survey. A majority (58%) of respondents rated their familiarity with NCDs at level 4 or higher on a 5-point Likert scale (Fig 1), and the most frequently identified conditions matched those cited in FGDs (Fig 2). Furthermore, 93.4% (n = 295) of student respondents agreed that NCDs represent a major health issue in Ghana, highlighting strong awareness of the national disease burden.

In addition to disease familiarity, students demonstrated a nuanced understanding of barriers to NCD care. Thematic analysis of FGDs revealed four core areas shaping these perceptions: limited awareness and understanding, socio-cultural influences, health system limitations, and financial constraints. Students frequently described community-level misconceptions about NCDs and emphasized how the asymptomatic nature of many conditions reduced urgency to seek care. Religious interpretations and cultural attitudes were also said to discourage early intervention. Structural challenges—including inadequate facilities, understaffing, and weak follow-up mechanisms—were consistently cited. Finally, the cost of diagnostic testing and chronic care emerged as a critical barrier, particularly for low-income households. S3 Table (in supplemental materials) summarizes these four themes and presents illustrative student quotes from FGDs.

### Interpretation

Students’ strong grasp of NCD challenges shows how deeply these conditions are woven into both their learning and daily life. Many spoke from firsthand exposure—through relatives, patients, or community service—linking theory to lived experience. Their ability to connect individual, social, and structural factors points to an emerging “systems awareness” early in their training. They recognize that NCD care is shaped not only by behavior but by the organization of Ghana’s health system, economic pressures, and public attitudes toward chronic illness. In this sense, their observations move beyond description: students are already diagnosing where the system falls short and imagining how it could respond more equitably.

### Key domain 2: students acceptance of SRFCs as an appropriate NCD intervention

Health professional students expressed strong interest in participating in a Student-Run Free Clinic (SRFC) focused on non-communicable disease (NCD) screening, early management, and self-care education. This interest was evident in focus group discussions and reinforced by survey findings, where 274 out of 316 respondents (86.7%) indicated willingness to participate in such an initiative.

Fisher-Freeman-Halton Exact Test was used to assess the association between academic program and willingness to participate, as some expected cell frequencies were below 5. There was no statistically significant association between willingness to participate and academic program (p = 0.0686). However, willingness to participate differed significantly according to stage of training (p = 0.0096). Clinical (non-final year) students demonstrated the highest willingness to participate (90.4%), followed by pre-clinical students (88.6%), while final-year students were less likely to express willingness to participate (75.4%). The original analysis of willingness according to academic year is provided in S5 Table.

However, logistic regression analysis identified familiarity with NCDs as a significant predictor of willingness to participate in SRFCs (β = 0.35, *p* = 0.007). Each unit increase in familiarity was associated with a 41% higher likelihood of participation (*aOR* = 1.41; 95% CI = 1.10–1.81). Model diagnostics confirmed adequate fit (Hosmer–Lemeshow χ² = 6.41, *p* = 0.39) and no multicollinearity (all VIF < 2). The model explained approximately 12% of the variance in willingness to participate (Nagelkerke R² = 0.12). These findings indicate that while enthusiasm for SRFCs was broadly shared across training programs, greater familiarity with NCDs independently increased students’ likelihood of engagement Table 3.

**Table 3:** Binary logistic regression examining familiarity with NCDs as a predictor of willingness to participate in SRFCs (*aOR* = 1.41; 95% CI = 1.10–1.81; *p* = 0.007). Model fit: Hosmer–Lemeshow χ² = 6.41, *p* = 0.39; Nagelkerke R² = 0.12.

Familiarity with NCDs vs. Willingness to Participate			
Parameter	Coefficient	P-value	Odds Ratio
Intercept	0.7058	0.1102	2.0254
Familiarity with NCDs	0.3454	0.0071	1.4126

**Fig 3:**
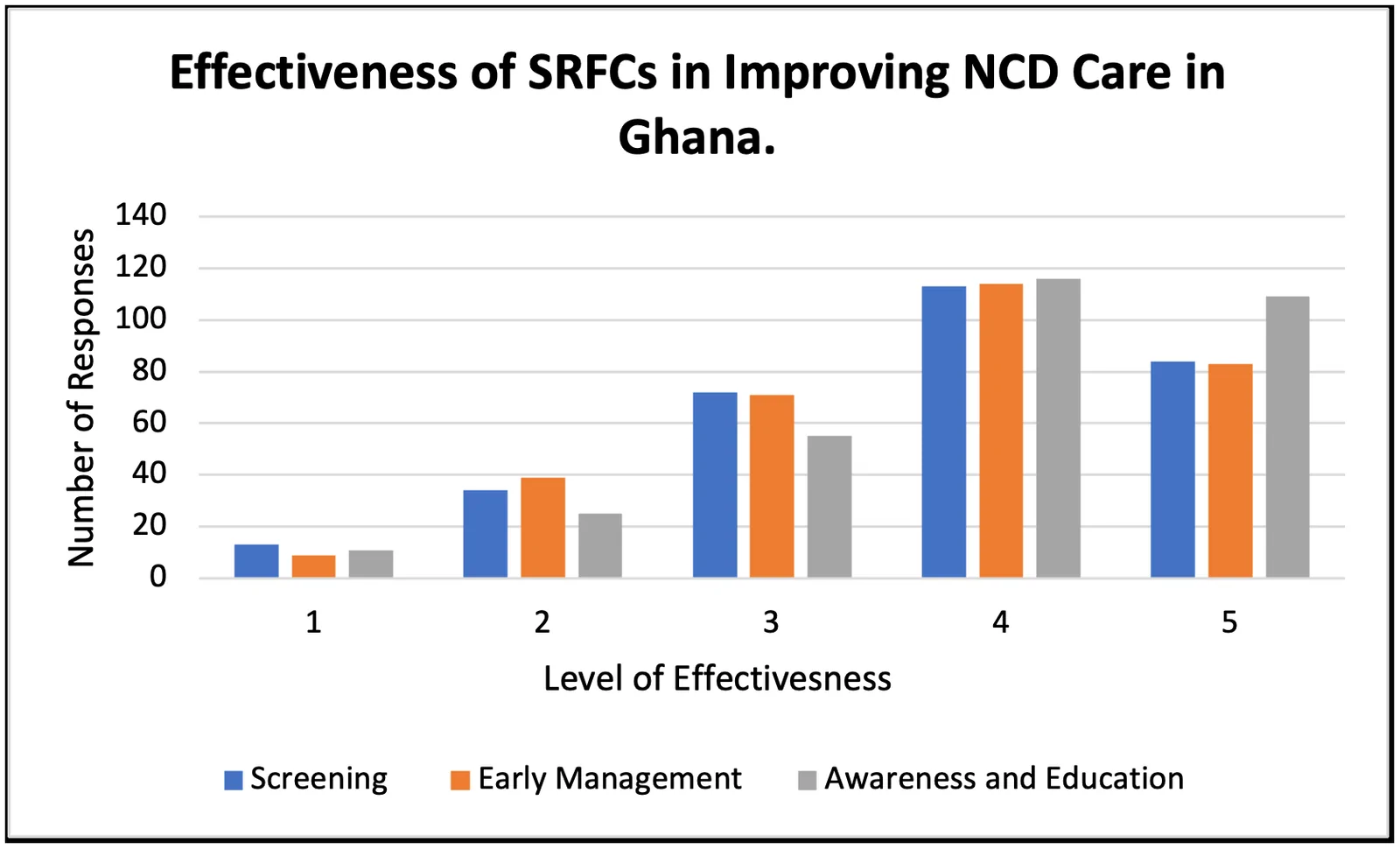
Health-professional students’ perceptions of the effectiveness of student-run free clinics (SRFCs) in improving non-communicable disease (NCD) care in Ghana. Composite bar chart showing students’ ratings of SRFC effectiveness in (blue) improving screening, (orange) enhancing early management, and (grey) strengthening awareness and education. Responses were rated on a five-point scale (1 = not effective; 5 = very effective).

Students’ strong willingness to participate was further supported by their perceptions of SRFCs as an effective intervention for addressing key barriers to NCD care. When asked to rate the effectiveness of SRFCs across screening, early management, and education on a 5-point scale, most respondents selected 4 or 5. Results are shown in Fig 3. Specifically, 197 students (62%) rated SRFCs as highly effective in improving NCD screening, and 225 students (71%) provided similar ratings for educational effectiveness, with 109 giving the maximum score. Perceptions of early management effectiveness also remained consistently high.

**Fig 4:**
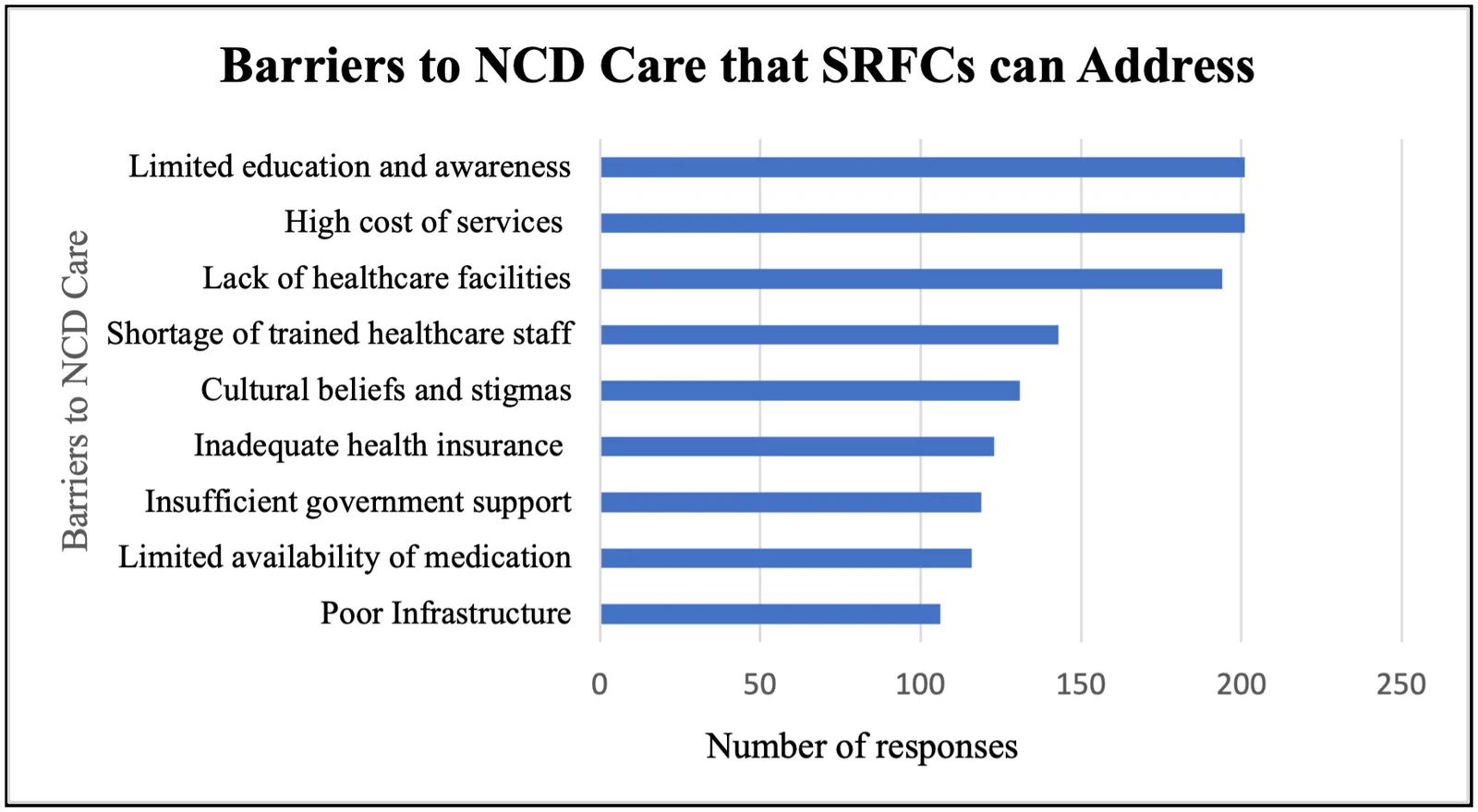
Barriers to NCD screening, early management, and self-care education that SRFCs can address.

These perceptions were further supported by students’ views on the specific barriers SRFCs are best positioned to address. As shown in Fig 4, students most frequently identified the high cost of healthcare services, lack of healthcare facilities, and limited education and awareness of NCDs as the top barriers SRFCs could effectively mitigate. Notably, these align directly with the top three barriers elicited earlier in the study (see S3 Table), suggesting a strong convergence between perceived need and perceived effectiveness.

### Interpretation

Although enthusiasm was observed across all training stages, final-year students expressed comparatively lower willingness to participate, likely reflecting competing academic and clinical responsibilities as graduation approaches. They view these clinics as a way to apply their training, strengthen clinical confidence, and live out their social responsibility. This alignment between service and education signals a shift toward a more socially accountable model of medical professionalism—one in which care for underserved populations is integral to learning, not an optional extra. Students’ belief that SRFCs could address real barriers shows a sense of ownership: they see themselves as active contributors to Ghana’s health-system reform rather than future employees waiting to be absorbed into it.

Focus group discussions provided deeper insights into the motivations driving student interest in SRFCs, as well as the challenges that might hinder participation. Students frequently cited their desire to serve communities and improve access to care for underserved populations. They also valued SRFCs as opportunities for hands-on learning, clinical skill development, and interprofessional collaboration. Some noted that logistical or symbolic incentives—such as mentorship, recognition, or refreshments—could further encourage participation, especially when balanced with academic demands.

However, students also identified notable barriers. The most frequently cited was academic workload, with concerns that participation could conflict with lectures, exams, or rotations. Students also raised concerns about the personal cost of transportation to clinic sites and the availability of essential materials. Finally, safety concerns—particularly about traveling to unfamiliar or insecure areas—were mentioned as a potential deterrent.

Yet their optimism is tempered by realism. Concerns about supervision, workload, cost, and safety highlight the limits of passion in a system stretched thin. Their caution reveals a clear understanding that sustainable volunteerism requires structure—faculty guidance, institutional support, and safeguards for student well-being. The coexistence of motivation and restraint illustrates a mature awareness of what it takes to turn good ideas into durable practice. S4 Table (in supplemental materials) summarizes motivating and discouraging factors influencing student’s willingness to participate in SRFCs with illustrative quotes

### Key domain 3: cross-sector endorsement of SRFCs as a contextually appropriate response to NCD care gaps

Stakeholders across the Ghana Health Service (GHS), university leadership, and community groups consistently endorsed Student-Run Free Clinics (SRFCs) as a relevant and timely intervention for addressing key gaps in non-communicable disease (NCD) care in Ghana. Despite representing distinct institutional and social roles, participants expressed overlapping motivations rooted in a shared commitment to expanding healthcare access, investing in student capacity, and strengthening partnerships between health systems, universities, and communities. This cross-sector endorsement was reflected across three convergent subdomains: alignment with institutional and community goals, confidence in students’ ability to deliver services, and recognition of SRFCs as valuable platforms for student training and interprofessional collaboration.

A. **Alignment with Institutional and Community Goals to Expand Access**

Stakeholders from GHS and universities emphasized that SRFCs align with their broader mandates to serve underserved communities and promote health equity. Faculty members framed SRFCs as natural extensions of existing outreach programs—such as the Community-Based Experience and Service (CoBES)—where students already engage in public education, disease monitoring, and referrals under supervision.

“UCC is known for its community service. For instance, at the School of Pharmacy, we do CoBES, where students go into the community and then, yes, render healthcare services. Some of the things they do is to educate the public, and again, to monitor some of these diseases, and do the referrals where appropriate. [] So, on our standards, we should encourage [any student-led initiative]” (UCF3).

GHS officials similarly viewed SRFCs as compatible with national health promotion efforts and existing community infrastructure, particularly the Wellness Clinics. They highlighted a foundation of goodwill between GHS and local communities that student-led efforts could readily tap into, noting that official endorsement would enhance credibility and facilitate community buy-in.

“When we mention our health promotion efforts, it’s necessary to mention that we love to collaborate. So, by all means, we’ll collaborate because one cannot do the work all alone. Students can partner with the Wellness Clinics and support the work we do. That will surely increase access for so many people.” (Ghana Health Service Official 2, GHS2)

“The Ghana Health Service freely goes out to areas like the rural settings and in fact the district zones... So already, the goodwill is there, all you need is to just tap into it.” (GHS4)

“The goodwill support is guaranteed. On our end, we can let the communities know that this clinic has our blessing and their universities’. Any push we can give, we will.” (GHS2)

Community members echoed this urgency and enthusiasm, describing SRFCs as critical responses to unmet needs in screening, education, and early management of NCDs.

“These diseases are killing us, but we don’t know. So, if “little doctor” can tell us, do this, don’t do that and they can explain the reason to us. It will help” (Community Reception Interview 2 Participant 3, CR2P3).

“When this initiative is established and we go for screening and some discoveries are uncovered, we will then be able to address it. But until then, we won’t know. We need this very urgently.” (CR1P1)

B. Confidence in Student Capacity and Community Trust.

Faculty members and GHS officials described students as capable contributors to public health outreach. They cited prior examples of student-led initiatives, like the annual “Health Weeks” where students lead several social and communal health initiatives, as evidence of their leadership and organizational potential. They emphasized that under appropriate supervision, students could extend NCD services and promote early detection through community engagement.

“We believe students can contribute to identifying these disease conditions early and also promote health awareness through health education, outreaches to let the community-dwelling citizens be aware that these are conditions that are mainly manageable and could prevent complications.” (UGF1.)

“We have a week set aside, we call it the health week, where students on their own are sent to different regions and different areas of the country. Usually, we come up with a theme, whether it’s Malaria prevention or hypertension prevention and then the students go out and contribute through community engagement activities in line with the theme of the year. (UGF2).

Students can help in the normal health screening like the BP check, [for] diabetes, the blood sugar check, they can do the fasting blood sugar checks and all those things”. (GHS3)

GHS personnel reinforced this confidence, noting that they already supervise trainees and would be willing to support student efforts in SRFCs through on-site mentorship and clinical guidance.

“We know these students already and we know what they can do. So, when they come, we can guide them with what they cannot do. With the simple tasks like blood glucose screening, BP checks, and the education, we can supervise them. We do that already.” (GHS1)

Community members also cited positive interactions with students in previous initiatives as a reason for their support. Many had built trust with students through past experiences and looked forward to future engagements. Some shared anecdotes of assisting students with tasks like setting up clinic canopies and spreading awareness through both official announcements and word of mouth. They appreciated students’ patience, attentiveness, and willingness to listen—qualities that made care feel truly patient-centered and fostered a strong sense of connection.

“Whenever these students come around, we help them assemble the canopies. They have time and take care of us, and this has been very helpful for us. Through this, we’ve been able to establish a sort of connection with these students as well.” (CR1P6)

C. Value for Student Training and Interprofessional Collaboration

University faculty highlighted that student-led initiatives provide essential hands-on training, enhancing clinical knowledge, patient interaction skills, and workforce readiness. They emphasized the alignment of student-led initiatives with interprofessional education goals, citing ongoing efforts to integrate interprofessional courses into the curriculum. They noted that students also recognized the benefits of collaboration, especially in community outreach.

“It is a very good idea. It is something everybody should embrace and it’s something we should all encourage. Last year, if I may remember, the College [of Health and Allied Sciences] started trying to put up interprofessional courses and programs. [] It’s something we are looking at and we know the advantages it will offer the students”. (UCF3).

“Yes, I was in a particular meeting for instance, and that meeting, I think the medical student brought up those ideas [for interprofessional collaboration]. They [went] to the community, and they realized that if the nurses were there, it would have been a great experience”. (UCF2).

Faculty also highlighted SRFCs as opportunities for junior students to learn from more advanced peers in real-world settings.

“We also believe that it’s an opportunity for some of our students in clinical years to also be there to understudy their senior colleagues.” (UCF2).

Community members also viewed the clinic as a crucial part of students’ education and were eager to support it. They recognized the importance of hands-on training in preparing students for future healthcare roles, understanding that these students would one day be responsible for their care.

“These students are coming to practicalize what they have been taught in taking care of us. And if these students are not given the requisite practical training, how then will they be able to effectively go about with their future career which entails taking care of us? So, for me, I am not bothered at all.” (CR1P3)

### Interpretation

The convergence of support among university leaders, Ghana Health Service officials, and community members suggests a moment of alignment across sectors that often work in isolation. Each group sees complementary benefits—better training, improved access, stronger trust—revealing shared readiness to collaborate. This unity hints at a broader appetite for innovation that bridges education and service. When viewed through a systems lens, SRFCs become more than a student project: they represent an emerging form of partnership governance where responsibility for health is shared among learners, institutions, and communities. Such alignment offers fertile ground for integrating SRFCs into national strategies for primary care and workforce development.

### Synthesis

Across these domains runs a consistent theme of readiness and shared purpose. Students bring energy and insight, institutions provide legitimacy and mentorship, and communities contribute trust and local knowledge. Together they outline the foundation for a model of care that is participatory, interprofessional, and contextually grounded. SRFCs thus hold promise not only for expanding NCD services but also for transforming how Ghana trains, supports, and connects its future health workforce with the populations they serve.

## Discussion

This study highlights broad stakeholder interest in student-led clinical interventions, such as SRFC, for NCD care in Ghana, emphasizing their potential to improve healthcare access in underserved communities. The findings align with existing literature on the role of SRFCs in expanding primary care services while simultaneously enhancing student training and interprofessional collaboration [10,11]. The strong familiarity with NCDs and their associated barriers among stakeholders underscores the relevance of such interventions, particularly in addressing gaps in early detection, management, and community education.

Institutional alignment further supports the feasibility of SRFCs, with university faculty and GHS officials recognizing their potential to complement existing health services. Notably, faculty emphasized that students have successfully participated in previous community health initiatives, reinforcing confidence in their ability to contribute meaningfully to NCD care. The strong student enthusiasm, with 86.7% expressing willingness to participate, underscores broad support for the concept; however, translating this energy into sustained impact will require a coordinating structure that can provide ongoing mentorship, institutional alignment, and systems for accountability and funding continuity. The identified opportunities for interprofessional collaboration are particularly promising, as interdisciplinary teamwork has been shown to improve health outcomes and enhance educational experiences.

An additional finding from the revised quantitative analysis was that willingness to participate varied according to stage of training. While willingness remained high across all groups, final-year students were significantly less likely to express interest in participating in SRFCs than pre-clinical and clinical (non-final year) students (75.4% vs. 88.6% and 90.4%, respectively). This finding may reflect the increased academic, clinical, licensing, and career-planning responsibilities faced by students nearing graduation, which can limit the time available for extracurricular service activities. Nevertheless, more than three-quarters of final-year students expressed willingness to participate, suggesting that SRFCs may still be able to engage senior trainees through flexible scheduling, leadership opportunities, and clearly defined educational roles.

This research contributes to the growing body of literature on SRFCs, particularly in low-resource settings like Ghana, where limited studies have explored their feasibility. While SRFCs are well-documented in high-income countries, particularly in the United States, their implementation in Sub-Saharan Africa remains underexplored [11,19]. Although SHAWCO at the University of Cape Town serves as a longstanding model, its establishment occurred under vastly different health and regulatory conditions [2]. Additionally, existing SRFC frameworks in high-income countries differ significantly from those in low-resource settings like Ghana. In high-income contexts, SRFCs are typically embedded within well-resourced academic institutions, benefit from clear supervisory structures, legal protections, and access to diagnostic tools. In contrast, Ghana faces limited clinical infrastructure, faculty shortages, and unclear policies on student-led care. These differences highlight the need for localized feasibility assessments to ensure safe and context-appropriate implementation.

The successful establishment of SRFCs in Ghana will depend on addressing several structural and regulatory challenges. Faculty and Ghana Health Service officials in this study emphasized the need for clear regulatory oversight to define students’ clinical scope and liability, as Ghana currently lacks formal policy frameworks governing student-led service. Faculty workload and supervision demands also pose constraints, as existing teaching responsibilities and limited faculty-to-student ratios may restrict sustained mentorship [20,21]. Financial sustainability further remains a concern: without recurring institutional or external funding, SRFCs risk short-term operation dependent on volunteer labor and donations [11]. Stakeholders also emphasized the importance of continuity of care, including mechanisms for patient follow-up, referral pathways, volunteer retention, and long-term institutional support, as essential components of a sustainable SRFC model for chronic disease management. Partnerships between universities, local GHS health facilities, and development partners could provide mechanisms for shared costs, supervision, and continuity.

Importantly, stakeholder support identified in this study should be interpreted as support for the broader SRFC concept rather than endorsement of a specific implementation model. Future work should focus on co-designing and evaluating contextually adapted SRFC frameworks that reflect Ghana’s regulatory environment, available resources, and health system priorities.

Establishing channels for collaboration and shared learning among emerging student-led clinics may further enhance accountability and quality improvement across sites. Recognizing and planning for these system-level considerations will be critical to translating enthusiasm into feasible, ethically sound, and sustainable SRFC models. In addition to structural partnerships, student-run clinics could benefit from reciprocal mentorship between established and emerging programs, both within Ghana and internationally. Such relationships would allow newer clinics to learn from mature programs’ experiences with supervision, logistics, and sustainability, while offering established clinics opportunities for innovation, cross-context collaboration, and shared evaluation of impact. This bidirectional exchange can promote adaptability on both sides—helping mature clinics refine their models while guiding new ones through implementation challenges. By transforming mentorship from a top-down transfer of knowledge into a shared process of reflection and co-development student-led clinics can evolve collectively, strengthening not only individual programs but also the broader ecosystem of socially accountable medical education [3,11].

By highlighting the role of interprofessional collaboration among students from various health disciplines, this study also builds on existing literature that emphasizes the importance of team-based care in managing complex conditions [22,23]. However, the literature often focuses on professionalized teams, and this research uniquely explores the feasibility of such collaboration at the student level. Despite the numerous advantages and push for interprofessional education (IPE) and training by organizations such as the Africa Interprofessional Education Network, IPE is still not well established in low- and middle-income countries, particularly across Sub-Saharan Africa, where the burden of disease is greater [24,25]. Thus, this study provides additional evidence for the interest and need for an interprofessional collaboration among health professional students. Additionally, the study’s focus on the community-based setting of SRFCs addresses a critical gap in understanding how such initiatives can be tailored to local contexts, where access to formal healthcare services is often limited.

### Implications for policy and practice

These findings provide valuable insights for policymakers, educators, and healthcare leaders considering the integration of student-led clinics into Ghana’s healthcare landscape. First, formalizing partnerships between universities, the GHS, and community health facilities could provide a structured framework for SRFCs, ensuring alignment with national health priorities. Importantly, stakeholder support identified in this study should be interpreted as support for the broader SRFC concept rather than endorsement of a specific implementation model. Future work should focus on co-designing and evaluating contextually adapted SRFC frameworks that reflect Ghana’s regulatory environment, available resources, and health system priorities. Finally, clear guidelines for interprofessional collaboration could further strengthen SRFCs, enabling students from different health disciplines to work together effectively in community settings.

### Strengths and weaknesses

This study offers several strengths, including its mixed-methods approach, which combines qualitative insights from key stakeholders with quantitative analysis of student perspectives. The inclusion of diverse voices—from faculty to community members—enhances the depth and applicability of the findings. Additionally, the study contributes novel data on the feasibility of SRFCs in Ghana, where limited research exists on this model.

However, some limitations should be acknowledged. The study relied on self-reported data, which may introduce social desirability bias, particularly regarding students’ willingness to participate. Because the quantitative survey predominantly sampled students from public universities, findings on student support for SRFCs may not be fully generalizable across all health professional training settings in Ghana. Another limitation is that participants were asked to evaluate the general concept of student-run free clinics rather than a fully specified Ghana-specific implementation model. As a result, stakeholder support may differ once operational details related to governance, supervision, funding, regulatory oversight, and scope of practice are defined.

Additionally, while the sample included key institutional, educational, health-system, and community stakeholders, direct participation from professional regulatory councils (e.g., Medical and Dental Council and Nursing and Midwifery Council) was not obtained. Their perspectives on governance, scope of practice, and regulatory oversight may further inform future implementation efforts. Future research should explore long-term implementation strategies, sustainability mechanisms, and the direct impact of SRFCs on health outcomes in Ghana.

## Conclusion

SRFCs present a promising model to address NCD care gaps in Ghana while simultaneously providing valuable training opportunities for health professional students. Strong institutional support, demonstrated student capacity, and community buy-in suggest that a high degree of feasibility of SRFC in this context. Future research should remain stakeholder-engaged and should explore contextual adaptation of the SRFC model with a focus on implementation strategies and potential funding options to support the model locally. In addition to site-level feasibility, future efforts may benefit from structured coordination across institutions and health authorities to promote consistency in supervision, reporting, and ethical standards. Such alignment could help sustain student-led activities while integrating them more effectively into Ghana’s primary care landscape.

## Supporting information

S1 Table Summary of coder agreement and validation procedures. This table outlines the analytic rigor steps used in qualitative analysis, including coder triangulation, ongoing calibration, member-checking, and reflexive documentation. Outcomes of each validation procedure are summarized.
(DOCX)

S2 Table Operational definitions of quantitative variables. This table provides definitions, measurement approaches, and coding structures for all variables included in the quantitative analysis (e.g., willingness to participate, familiarity with NCDs, academic program/level, perceived feasibility/support).
(DOCX)

S3 Table Student perspectives on barriers to NCD care. This table summarizes key qualitative themes and illustrative quotes from student focus group discussions, including limited awareness of NCDs, sociocultural barriers, health system limitations, and financial constraints.
(DOCX)

S4 Table Factors influencing students’ willingness to participate in student-run free clinics. This table presents motivating and discouraging factors for SRFC participation, supported by representative quotes. Motivating factors include community impact, professional development, and interprofessional collaboration; discouraging factors include academic conflicts, resource limitations, and safety concerns.
(DOCX)

S5 Table Willingness to participate in student-run free clinics (SRFCs) by academic level among health-professional students. This table presents willingness to participate in SRFCs according to academic level (Years 1–6) across all surveyed health-professional disciplines. Results are reported as frequencies and percentages of students expressing willingness to participate within each academic level and provide the original academic-level analysis used prior to reclassification by stage of training.
(DOCX)

S1 Appendix List of abbreviations. This appendix provides definitions of abbreviations used throughout the manuscript, including NHIS, CHPS, NCDs, CVDs, SRFC, SHAWCO, and others.
(DOCX)
