# Supplementary material for: Exploring stakeholder support for student-run free clinics to address gaps in non-communicable disease care in Ghana: An exploratory sequential mixed-methods study
Source: PLOS Glob Public Health. 2026 Sep 21;6(9):e0005092. doi: 10.1371/journal.pgph.0005092 (PMC13592639; doi:10.1371/journal.pgph.0005092)
Supplement: Supplementary file 1 [file pgph.0005092.s001.docx]

| **Quality Criterion** | **Procedure** | **Evidence/Outcome** |
| --- | --- | --- |
| Coder triangulation | Two analysts independently coded first 20% of transcripts; discussed differences and refined codebook | Reached full consensus on all major codes; differences resolved through discussion |
| Ongoing calibration | Periodic double-coding of 20% of transcripts; review of excerpts at each analytic round | Codebook remained stable; no major reclassification after second calibration |
| Member-checking | Shared one-page thematic summary with 13 participants (students = 6; faculty/GHS = 3; community = 4) | Participants confirmed overall accuracy and added emphasis on transport cost and safety |
| Reflexivity and audit | Maintained brief notes on analytic decisions and rationale | Enhanced transparency and dependability |

**S1 Table.** Summary of Coder Agreement and Validation Procedures. (Note: No formal coefficient such as Cohen’s κ was calculated; agreement was determined through consensus and documented discussion.)
