# Supplementary material for: Exploring stakeholder support for student-run free clinics to address gaps in non-communicable disease care in Ghana: An exploratory sequential mixed-methods study
Source: PLOS Glob Public Health. 2026 Sep 21;6(9):e0005092. doi: 10.1371/journal.pgph.0005092 (PMC13592639; doi:10.1371/journal.pgph.0005092)
Supplement: Supplementary file 2 [file pgph.0005092.s002.docx]

| **Variable** | **Operational Definition** | **Measurement / Coding** | **Type** |
| --- | --- | --- | --- |
| Willingness to participate in SRFCs | Indicates whether a student expressed readiness to engage in student-run free clinic activities for NCD care. | Binary: 1 = “Yes,” 0 = “No.” | Dependent (Outcome) |
| Familiarity with NCDs | Self-reported understanding of non-communicable diseases, their management, and related healthcare challenges. | Rated on a 5-point Likert scale (1 = “Not familiar,” 5 = “Very familiar”). | Independent |
| Prior community outreach experience | Participation in health-related outreach or volunteer activities during training. | Binary: 1 = “Yes,” 0 = “No.” | Independent |
| Academic program | Field of study within health professions education. | Categorical: Medicine, Nursing, Pharmacy, Optometry, Allied Health Sciences. | Independent |
| Academic level | Current year of study in academic program. | Ordinal: 1st, 2nd, 3rd, 4th, 5th or 6th year. | Independent |
| Gender | Self-identified gender of respondent. | Binary: 1 = “Male,” 0 = “Female.” | Control |
| Perceived institutional support | Extent to which respondents believe their institution supports student-led interventions. | 5-point Likert scale (1 = “Strongly disagree,” 5 = “Strongly agree”). | Independent |
| Perceived feasibility of SRFCs | Perception of how realistic or implementable SRFCs would be within their institutional context. | 5-point Likert scale (1 = “Not feasible,” 5 = “Highly feasible”). | Independent |

**S2 Table**. Operational Definitions of Quantitative Variables
