# Supplementary material for: Exploring stakeholder support for student-run free clinics to address gaps in non-communicable disease care in Ghana: An exploratory sequential mixed-methods study
Source: PLOS Glob Public Health. 2026 Sep 21;6(9):e0005092. doi: 10.1371/journal.pgph.0005092 (PMC13592639; doi:10.1371/journal.pgph.0005092)
Supplement: Supplementary file 3 [file pgph.0005092.s003.docx]

**Summary of Student Perspectives on Barriers to NCD Care.**

| **Themes from FDGS** | **Illustrative Student Quotes** |
| --- | --- |
| **Limited Awareness and Understanding of NCDs** | *“Most of the market [women], when their head is aching, they just walk in [to a drug store] and they get [paracetamol]... that headache could be anything.” (UG2S2)*  *“Sometimes they know, but they don’t know much… they just know the surface like ‘I have high blood pressure.’” (UG1S3)* |
| **Socio-Cultural Barriers** | *“Some people are strongly attached to their religious affiliations… their pastors tell them, ‘As a child of God, the Holy Spirit will heal you.’” (UG2S6)*  *“They are indifferent about it… they say, ‘Is it because we will die that we won’t sleep?’” (UC3S3)* |
| **Health System Limitations** | *“Even if they have a CHPS compound, they don’t have health professionals there… just one nurse or something.” (UC3S2)*  *“The follow-up system in Ghana is not proper… sometimes they call once or twice and give up.” (UG1S3)* |
| **Financial Constraints** | *“Most people would not prefer to visit the hospitals because of the rate at which bills are being increased.” (UC2S3)*  *“Let’s say the drug costs 50 cedis, but the person earns only 5 cedis a day… and came with just 10 cedis. How do you expect them to buy it?” (UC1S2)* |

**S3 Table.** Summary of Student Perspectives on Barriers to NCD Care.
