# Supplementary material for: Exploring stakeholder support for student-run free clinics to address gaps in non-communicable disease care in Ghana: An exploratory sequential mixed-methods study
Source: PLOS Glob Public Health. 2026 Sep 21;6(9):e0005092. doi: 10.1371/journal.pgph.0005092 (PMC13592639; doi:10.1371/journal.pgph.0005092)
Supplement: Supplementary file 4 [file pgph.0005092.s004.docx]

**Motivating and Discouraging Factors Influencing Students’ Willingness to Participate in SRFCs.**

| **Category** | **Theme** | **Illustrative Quotes** |
| --- | --- | --- |
| Motivating Factors | Community and Social Impact | Yeah, I absolutely would. It's great to give back to the community. And it looks good on the resume. It gives me experience in the clinic as well.” (UG2S5) |
|  | Academic and Professional Development | “Personally, I would also join if there is an opportunity because one, it will help me to learn and two, help me to build connections, not only with the health professionals but also with the students and [] the patients that will come.” (UC2S4).  “I think it's a great opportunity that should be implemented.[] it gives us another opportunity to further our skills.” (UC2S1). |
|  | Opportunities for Interprofessional Collaboration | “I have not gotten the chance to I mean attend program with different health professionals. So even seeing that people from different health areas are coming together for such a program like this, I think it will be wise to be a part of this so that we all share knowledge and learn from each other” (UC1S7). |
|  | Incentives | “Students want ‘item 13’ [refreshments] and T-shirts. So, if you provide that, I will keep showing up”. (UG3S2). |
| Discouraging Factors | Academic Obligations and Conflicts | “Okay, for me personally, if it doesn’t clash with any of my academic activities, why not? Because I am enthused about helping.” (UC2S1)  “I feel I’ll be interested but maybe like I was saying, if it’s going to against my academics or I’m going to get stressed out or something, I’m sure I’ll drop out at some point”. (UG1S3) |
|  | Resource Demands | If there is no means of transport and I will have to spend to go there, I won't be that happy to go for the thing. I will be reluctant. As well as availability of resources to work with” (UC2S7) |
|  | Safety Concerns | “I will also say the security. [] I wouldn't go to a place where I know they can attack me anytime. Certain areas when you go there you know they can attack you” (UC2S5). |

**S4 Table.** Summary of qualitative themes and illustrative quotes from focus group discussions (FGDs) with health-professional students regarding factors that motivate or discourage participation in student-run free clinics. Motivating factors included community impact, professional development, interprofessional collaboration, and incentives. Discouraging factors centered on academic workload, resource limitations, and safety concerns.
