# Supplementary material for: Exploring stakeholder support for student-run free clinics to address gaps in non-communicable disease care in Ghana: An exploratory sequential mixed-methods study
Source: PLOS Glob Public Health. 2026 Sep 21;6(9):e0005092. doi: 10.1371/journal.pgph.0005092 (PMC13592639; doi:10.1371/journal.pgph.0005092)
Supplement: Supplementary file 5 [file pgph.0005092.s005.docx]

| **Academic Level** | 1st Year | 63 (86.3) | 10 (13.7) | **0.58** |
| --- | --- | --- | --- | --- |
|  | 2nd Year | 57 (87.7) | 8 (12.3) |  |
|  | 3rd Year | 34 (87.2) | 5 (12.8) |  |
|  | 4th Year | 56 (87.5) | 8 (12.5) |  |
|  | 5th Year | 55 (88.7) | 7 (11.3) |  |
|  | 6th Year | 9 (69.2) | 4 (30.8) |  |

**S5 Table.** Willingness to participate in student-run free clinics (SRFCs) by academic level among health-professional students. This table presents the original analysis of willingness to participate in SRFCs according to academic level (Years 1–6) across all surveyed disciplines. Results are shown as frequencies and percentages of students expressing willingness to participate in SRFCs within each academic level. This analysis is provided as supplementary material for transparency, while the primary manuscript presents willingness according to stage of training (pre-clinical, clinical non-final year, and final year) to account for differences in program duration across disciplines.
