# Supplementary material for: Exploring stakeholder support for student-run free clinics to address gaps in non-communicable disease care in Ghana: An exploratory sequential mixed-methods study
Source: PLOS Glob Public Health. 2026 Sep 21;6(9):e0005092. doi: 10.1371/journal.pgph.0005092 (PMC13592639; doi:10.1371/journal.pgph.0005092)
Supplement: Supplementary file 6 [file pgph.0005092.s006.docx]

**S1 Appendix**

**List of Abbreviations**

CHOs – Community Health Officers

CHPs – Community-based Health Planning and Services

CVDs – Cardiovascular Diseases

NCD – Non-Communicable Diseases

NHIS - National Health Insurance Scheme

NOP – Networks of Practice

NPHWs - Non-Physician Healthcare Providers (NPHWs)

SHAWCO - Students' Health and Welfare Centers Organization

SRFC – Student Run Free Clinic
