# Supplementary material for: Exploring stakeholder support for student-run free clinics to address gaps in non-communicable disease care in Ghana: An exploratory sequential mixed-methods study
Source: PLOS Glob Public Health. 2026 Sep 21;6(9):e0005092. doi: 10.1371/journal.pgph.0005092 (PMC13592639; doi:10.1371/journal.pgph.0005092)
Supplement: Supplementary file 7 [file pgph.0005092.s008.docx]

**Response to Reviewers**

**Manuscript Title:** *Exploring Stakeholder Support for Student-Run Free Clinics to Address Gaps in Non-Communicable Disease Care in Ghana: An Exploratory Sequential Mixed-Methods Study*
**Journal:** *PLOS Global Public Health*
**Corresponding Author:** Brian A. Fleischer (B.A.F.)

We sincerely thank the editor and reviewer for their constructive and thoughtful feedback, which greatly strengthened the quality, clarity, and rigor of our manuscript. The revised version incorporates expanded methodological details, improved analytic justification, clearer reporting of quantitative and qualitative results, and streamlined tables and figures. Below, we provide detailed, point-by-point responses to all reviewer comments.

### Reviewer Comments and Author Responses

#### **1.** Reviewer comment: Purposive sampling in the qualitative phase is appropriate, but inclusion criteria for key informants and community members need clearer justification. Explain how saturation was determined. Thematic analysis appears descriptive rather than interpretive. Member-checking and coder triangulation are mentioned but not evidenced. Include a summary table or description of inter-coder reliability (e.g., Cohen’s κ).

**Author response:**
We appreciate this valuable feedback and made several changes to enhance analytic transparency and rigor:

- **Inclusion criteria:** Now clearly defined for all participant groups (students, faculty, Ghana Health Service officials, and community members) in *Methods (pp. 6–7)*. Vulnerable groups were excluded to ensure ethical compliance.
- **Saturation:** Added clarification that thematic saturation was reached after six FGDs with students, six KIIs, and two community FGDs, when no new codes emerged.
- **Interpretive approach:** Reframed the analysis as *interpretive thematic analysis*, emphasizing underlying meanings beyond surface description.
- **Coder triangulation:** Two analysts coded a subset of transcripts independently, discussed discrepancies, and refined the codebook iteratively. Approximately 20% of transcripts were double-coded for calibration. While Cohen’s κ was not computed, intercoder agreement was achieved through consensus and audit review.
- **Member checking:** Conducted with 14 participants who reviewed and validated the thematic summaries.
- **Supplementary evidence:** *Supplementary Table S1* now includes the final coding framework and major themes; *Table 2* provides illustrative participant quotes.

#### **2.**Reviewer comment:Clarify whether survey items derived from qualitative themes underwent pilot validation, and include evidence of reliability (e.g., Cronbach’s alpha, content and face validity). The sample of 316 students is sizable, but the sample size calculation is not reported. Authors should specify assumptions and formula used to derive this number (expected effect size, power, confidence level). The logistic regression results (OR = 1.41, p = 0.007) are presented without model fit indices, confidence intervals, or assumption testing. Include 95% CIs, and specify checks for multicollinearity and goodness-of-fit (e.g., Hosmer–Lemeshow test). State whether Chi-square assumptions (expected frequencies ≥ 5) were verified, given the relatively small subgroup counts. A re-analysis using Fisher’s Exact Test is warranted if those assumptions were not met.

**Author response:**
We strengthened the quantitative reporting as follows:

- **Survey validation:** Clarified that survey items were derived from qualitative findings and reviewed by content experts for construct and face validity. As stated: *“Given the categorical and exploratory nature of the items, formal reliability statistics such as Cronbach’s α were not calculated; however, item coherence was confirmed through internal review of response consistency and expert agreement on construct alignment.”*
- **Questionnaire structure:** The survey included 12 items covering demographics, prior exposure to student-led interventions, perceived barriers/enablers, and willingness to participate.
- **Sample size:** Now described using the **Yamane formula** with assumptions of 95% confidence and 5% margin of error (n = 384 target, n = 316 achieved).
- **Statistical assumptions:** Chi-square assumptions were verified; Fisher’s Exact Test was used where expected frequencies were < 5.
- **Model diagnostics:** Added model fit indices and diagnostics: Hosmer–Lemeshow χ² = 6.41 (p = 0.39), Nagelkerke R² = 0.12, and multicollinearity check (VIF < 2).
- **Results:** Updated to report 95% CIs: *Familiarity with NCDs significantly predicted willingness to participate (aOR = 1.41; 95% CI = 1.10–1.81; p = 0.007).*
- **Operational definitions:** Now included in *Supplementary Table S2*, detailing variable names, coding, and measurement types.

#### **3.** Reviewer comment:Clarify whether Chi-square tests assessed associations or differences.

**Author response:**
We clarified in *Methods (p. 9)* that Chi-square and Fisher-Freeman-Halton Exact Tests were used to test **associations** between categorical variables, rather than differences in means or proportions.

#### **4.** Reviewer comment:Include a list of operational definitions for quantitative variables.

**Author response:**
All operational definitions are now included in *Supplementary Table S2*, specifying coding, measurement scale, and data type for each quantitative variable (e.g., *Familiarity with NCDs*, *Willingness to participate*). This structure maintains clarity while reducing redundancy in the main text.

#### **5.** Reviewer comment:The discussion should address structural barriers such as regulatory oversight, sustainable funding, and faculty load, supported by citations.

**Author response:**
We revised the *Discussion (pp. 16–18)* to temper generalizations and integrate a stronger critical reflection on implementation feasibility:

- Expanded on **structural barriers** including regulatory oversight, limited faculty capacity, and sustainability of funding models.
- Added new citations — **Awiagah et al. (2024)** and **Ziba et al. (2021)** — to contextualize faculty supervision constraints and institutional challenges in Ghana.
- Introduced the concept of **reciprocal mentorship** between established and emerging SRFCs, highlighting bidirectional learning and adaptation across institutions without referencing specific programs.

#### **6.** Reviewer comment:Figures 3–6 depict near-identical rating scales; consider combining or summarizing. Tables 3–5 contain redundant information; table numbering should be corrected.

**Author response:**
We streamlined visuals and tabular data to enhance readability:

- **Figures 3–5** were merged into a composite **Figure 3** with subpanels (a–c) illustrating SRFC effectiveness in improving screening, early management, and education.
- **Tables 3–5** were condensed into two tables summarizing bivariate associations and the logistic regression model. - Extended results and descriptive data were moved to *Supplementary Tables S1–S4*.
- Table and figure numbering were corrected throughout.

### **Conclusion**

We thank the reviewer and editorial team for their insightful feedback. The revised manuscript now provides greater methodological transparency, interpretive depth, and policy relevance. We believe these revisions fully address all concerns and bring the manuscript in line with *PLOS Global Public Health* standards.

Respectfully,
**Brian A. Fleischer (B.A.F.)**
Corresponding Author
