# Supplementary material for: Exploring stakeholder support for student-run free clinics to address gaps in non-communicable disease care in Ghana: An exploratory sequential mixed-methods study
Source: PLOS Glob Public Health. 2026 Sep 21;6(9):e0005092. doi: 10.1371/journal.pgph.0005092 (PMC13592639; doi:10.1371/journal.pgph.0005092)
Supplement: Supplementary file 8 [file pgph.0005092.s009.docx]

**Student -Run Free Clinic (SRFC) Study Review**

**I would like to raise the following issues with the authors:**

- Were the participants familiar with the S-RFCs concept prior to their involvement in this study. If not, how was that achieved?
- The S-RFC models are highly contextual in nature because of, among others, the regulatory and the resources requirements. If a Ghana-relevant model is conceptualized and presented to the same participants, could that change the outcome of this study?
- Were there representative from the professions regulating councils, e.g. Nursing Council, Medical Council, etc participating in this study?
- Were all the community members participating in this study NCDs patients themselves and from the underserved communities i.e. potential users of the S-RFCs services?
- The question about the NCDs familiarity in the quantitative study is non-specific. Does it involve:
  - General knowledge about NCDs.
  - Familiarity with the burden of NCDs in Ghana?
  - Familiarity with NCDs prevention and screening?.
  - Familiarity with early management of NCDs.
- Table (2) summarizes the willingness to participate in relation to the academic year of study. Since the training programmes differ in length across the disciplines and we anticipate the students role in the S-RFCs will be linked to their academic level, the data should be analysed by the academic level rather than year of study e.g. the willingness to participate among the final-year students across all disciplines.

- Since continuity of care is a critical component of NCDs services, it could have been great if sustainability challenges were raised during the FGDs / KIIs phase of the study.
