# Supplementary material for: Exploring stakeholder support for student-run free clinics to address gaps in non-communicable disease care in Ghana: An exploratory sequential mixed-methods study
Source: PLOS Glob Public Health. 2026 Sep 21;6(9):e0005092. doi: 10.1371/journal.pgph.0005092 (PMC13592639; doi:10.1371/journal.pgph.0005092)
Supplement: Supplementary file 9 [file pgph.0005092.s010.docx]

**Response to Reviewer #2**

We sincerely thank the reviewer for their thoughtful feedback and for recognizing the overall quality and rigor of the revised manuscript. We have addressed the remaining comment as follows:

**Comment:**
*The quantitative survey heavily sampled students from public universities, with very limited representation from private institutions. This limits the generalizability of findings on student support for SRFCs. The manuscript would benefit from its mention in limitation section.*

**Response:**
Thank you for highlighting this important point. We agree that the predominance of survey respondents from public universities limits the generalizability of findings to students in private institutions. We have now explicitly acknowledged this in the limitations section of the Discussion.

**Revision made in manuscript:**
We added the following sentence to the “Strengths and Weaknesses” subsection of the Discussion:
“Because the quantitative survey predominantly sampled students from public universities, with limited representation from private institutions, findings on student support for SRFCs may not be fully generalizable across all health professional training settings in Ghana.”
