# Supplementary material for: Exploring stakeholder support for student-run free clinics to address gaps in non-communicable disease care in Ghana: An exploratory sequential mixed-methods study
Source: PLOS Glob Public Health. 2026 Sep 21;6(9):e0005092. doi: 10.1371/journal.pgph.0005092 (PMC13592639; doi:10.1371/journal.pgph.0005092)
Supplement: Supplementary file 10 [file pgph.0005092.s011.docx]

**Response to Reviewer**

We sincerely thank the reviewer for their careful reading of our manuscript, “Exploring Stakeholder Support for Student-Run Free Clinics to Address Gaps in Non-Communicable Disease Care in Ghana: An Exploratory Sequential Mixed-Methods Study,” and for their thoughtful and constructive comments. We greatly appreciate the opportunity to revise the manuscript and believe that the reviewer’s suggestions have strengthened the clarity, rigor, and interpretation of our findings.

Below, we provide a point-by-point response to each comment. All corresponding revisions have been incorporated into the revised manuscript and supplementary materials.

**Reviewer Comment 1:**

*Were the participants familiar with the S-RFCs concept prior to their involvement in this study? If not, how was that achieved?*

**Response:**

We thank the reviewer for this important observation. While some participants had prior exposure to student-led health outreach activities, familiarity with the formal Student-Run Free Clinic (SRFC) model was not assumed. To ensure a common understanding across participants, moderators provided a standardized explanation of the SRFC concept at the beginning of each focus group discussion and key informant interview. This explanation included the purpose and structure of SRFCs, examples of established programs in both the United States and Africa—including HAVEN Free Clinic at Yale University and SHAWCO at the University of Cape Town—the roles and responsibilities of health professional students, limits of student practice, faculty supervision requirements, and the regulatory oversight mechanisms typically used to ensure patient safety. Participants were encouraged to ask clarifying questions before discussing the feasibility and acceptability of SRFCs in the Ghanaian context.

Similarly, survey respondents were provided with a brief standardized description of the SRFC model before answering questions related to willingness to participate and perceived effectiveness. This description outlined the clinic's focus on non-communicable disease screening, early management, and health education, as well as the role of health professional students working under appropriate supervision.

We have now clarified this procedure in the Methods section of the manuscript.

**Reviewer Comment 2:**

*The S-RFC models are highly contextual in nature because of, among others, the regulatory and the resources requirements. If a Ghana-relevant model is conceptualized and presented to the same participants, could that change the outcome of this study?*

**Response:**

We thank the reviewer for this thoughtful observation and agree that student-run free clinic (SRFC) models are highly contextual and must be adapted to local regulatory, educational, and resource environments. The objective of the present study was exploratory in nature and focused on assessing stakeholder perceptions regarding the feasibility, acceptability, and potential value of SRFCs in Ghana, rather than evaluating a predefined implementation model.

To address this, participants were encouraged during interviews and focus group discussions to reflect on how an SRFC should be adapted to the Ghanaian context, including considerations related to supervision, scope of practice, institutional partnerships, sustainability, logistics, and regulatory oversight. These discussions generated many of the recommendations that informed our interpretation of feasibility and support.

We acknowledge that stakeholder perceptions may evolve when presented with a specific operational model and that support for particular design features may vary depending on implementation details. We have therefore added a statement to the Discussion and Limitations sections acknowledging that stakeholder responses were based on the SRFC concept rather than a fully developed Ghana-specific model. Development and evaluation of a contextually adapted Ghanaian SRFC framework informed by stakeholder recommendations is the focus of ongoing work and will be reported separately.

**Reviewer Comment 3:**

*Were there representatives from the professional regulating councils, e.g., Nursing Council, Medical Council, etc., participating in this study?*

**Response:**

We thank the reviewer for this important question. Formal representation from the professional regulatory councils was not included among the study participants. However, several faculty members and senior Ghana Health Service (GHS) officials who participated in key informant interviews had experience working with, advising, or serving on relevant professional regulatory bodies and were therefore able to provide informed perspectives regarding issues of professional regulation, supervision, scope of practice, and student involvement in service delivery.

We also attempted to engage relevant regulatory councils during the stakeholder recruitment process; however, we did not receive formal responses within the study period. We acknowledge that direct participation from regulatory councils would have provided an additional perspective on governance and implementation considerations and have added this point as a limitation of the study.

**Reviewer Comment 4:**

*Were all the community members participating in this study NCD patients themselves and from the underserved communities, i.e., potential users of the S-RFCs services?*

**Response:**

We thank the reviewer for this important question. All community participants were recruited from underserved communities within the catchment areas of the study sites and were selected because they represented potential beneficiaries of services that could be offered through a student-run free clinic (SRFC), including NCD screening, health education, referral, and early management support.

While not all community participants were individuals living with non-communicable diseases (NCDs), several reported a current or previous history of NCDs or described experiences caring for family members affected by conditions such as hypertension and diabetes. The study intentionally sought a broader community perspective, as attitudes toward healthcare access, preventive screening, and community-based interventions are shaped not only by individuals living with NCDs but also by family members and community stakeholders who influence health-seeking behaviors.

To improve clarity, we have revised the Methods section to further describe the characteristics and rationale for inclusion of community participants.

**Reviewer Comment 5:**

*The question about NCD familiarity in the quantitative study is non-specific. Does it involve:
• General knowledge about NCDs?
• Familiarity with the burden of NCDs in Ghana?
• Familiarity with NCD prevention and screening?
• Familiarity with early management of NCDs?*

**Response:**

We thank the reviewer for this important observation. In the quantitative survey, “familiarity with NCDs” was assessed using a self-reported 5-point Likert scale asking students to rate their overall familiarity with non-communicable diseases. This measure was intended to capture broad perceived familiarity rather than a specific domain of NCD knowledge.

However, the survey also included related items asking students to identify common NCDs and select perceived barriers to NCD screening, early management, and self-care education in Ghana. These additional items provided contextual support for interpreting the familiarity measure, as students commonly identified hypertension, diabetes, stroke, and cancer as major NCDs and recognized barriers such as high cost of care, limited health facilities, and low awareness/education. Therefore, we interpret the familiarity variable as reflecting general awareness and exposure to NCDs and NCD-related care barriers, rather than specific knowledge of disease burden, prevention, screening, or clinical management.

We agree that the original wording may have been insufficiently specific and have revised the Methods and Results sections to clarify how NCD familiarity was measured and interpreted. We have also added a limitation noting that the study did not use a validated multidimensional NCD knowledge scale capable of distinguishing between familiarity with disease burden, prevention, screening, and management.

**Reviewer Comment 6:**

*Table (2) summarizes the willingness to participate in relation to the academic year of study. Since the training programmes differ in length across the disciplines and we anticipate the students’ role in the S-RFCs will be linked to their academic level, the data should be analysed by academic level rather than year of study, e.g., the willingness to participate among the final-year students across all disciplines.*

**Response:**

We thank the reviewer for this valuable suggestion. We agree that programme duration varies across disciplines and that students’ anticipated roles within student-run free clinics (SRFCs) may be more closely related to stage of training than academic year alone. Accordingly, we reclassified students into pre-clinical, clinical (non-final year), and final-year categories using discipline-specific curricular structures and repeated the analysis.

For Medicine and Pharmacy (6-year programmes), Years 1–3 were classified as pre-clinical, Years 4–5 as clinical (non-final year), and Year 6 as final year. For all other programmes, Year 1 was classified as pre-clinical, Years 2–3 as clinical (non-final year), and Year 4 as final year.

The revised analysis demonstrated a statistically significant association between stage of training and willingness to participate in SRFCs (p = 0.0096). Clinical (non-final year) students demonstrated the highest willingness to participate (90.4%), followed by pre-clinical students (88.6%), while final-year students were comparatively less likely to express willingness to participate (75.4%). We have revised Table 2 to present this more educationally meaningful categorization and moved the original academic-level analysis to Supplementary Table S5 for transparency.

**Reviewer Comment 7:**

*Since continuity of care is a critical component of NCD services, it could have been great if sustainability challenges were raised during the FGDs/KIIs phase of the study.*

**Response:**

We thank the reviewer for this important observation and agree that sustainability and continuity of care are critical considerations for any student-run free clinic (SRFC) model focused on non-communicable diseases (NCDs). Sustainability-related issues were in fact explored during both the focus group discussions and key informant interviews. Participants across stakeholder groups—including students, faculty, Ghana Health Service officials, and community members—identified several challenges related to long-term implementation, including faculty supervision capacity, funding, volunteer retention, logistical support, institutional commitment, and mechanisms for continuity of care and patient follow-up.

While the present manuscript focuses primarily on stakeholder support, feasibility, and acceptability of the SRFC concept, several sustainability considerations are discussed in the manuscript, particularly within the Discussion section addressing regulatory oversight, faculty workload, partnerships, and financial sustainability. A more detailed thematic analysis focused specifically on sustainability challenges and stakeholder recommendations for long-term implementation is being prepared as a separate manuscript. We have clarified in the Discussion that sustainability emerged as an important consideration during stakeholder consultations and warrants further investigation.

Once again, we thank the reviewer for their thoughtful comments and valuable suggestions. We believe that the revisions made in response to these comments have improved the clarity, methodological transparency, and overall quality of the manuscript. We appreciate the opportunity to revise our work and respectfully submit the revised manuscript for further consideration.
