# Supplementary material for: Exploring stakeholder support for student-run free clinics to address gaps in non-communicable disease care in Ghana: An exploratory sequential mixed-methods study
Source: PLOS Glob Public Health. 2026 Sep 21;6(9):e0005092. doi: 10.1371/journal.pgph.0005092 (PMC13592639; doi:10.1371/journal.pgph.0005092)
Supplement: Peer review history

# Peer review: Decision Letter 0

**DOI:** 10.1371/journal.pgph.0005092.r001

**Authors:** Katherine Kokkinias

2 Oct 2025

PGPH-D-25-02203

Exploring Stakeholder Support for Student-Run Free Clinics to Address Gaps in Non-Communicable Disease Care in Ghana: An Exploratory Sequential Mixed-Methods Study.

PLOS Global Public Health

Dear Dr. Fleischer,

Thank you for submitting your manuscript to PLOS Global Public Health. After careful consideration, we feel that it has merit but does not fully meet PLOS Global Public Health’s publication criteria as it currently stands. Therefore, we invite you to submit a revised version of the manuscript that addresses the points raised during the review process.

==============================

Please note that we have only been able to secure a single reviewer to assess your manuscript. We are issuing a decision on your manuscript at this point to prevent further delays in the evaluation of your manuscript. Please be aware that the editor who handles your revised manuscript might find it necessary to invite additional reviewers to assess this work once the revised manuscript is submitted. However, we will aim to proceed on the basis of this single review if possible.

The reviewer raised a number of concerns that need attention. They request additional information on methodological aspects of the study (such as the how saturation was determined, if intercoder reliability was calculated, sample size calculations for the surveys, and statistical assumptions). The reviewer also suggested tempering the conclusions regarding the generalizability of the study and describing implementation challenges.

Could you please revise the manuscript to carefully address the concerns raised?

==============================

Please submit your revised manuscript by Nov 16 2025 11:59PM. If you will need more time than this to complete your revisions, please reply to this message or contact the journal office at globalpubhealth@plos.org. When you're ready to submit your revision, log on to https://www.editorialmanager.com/pgph/ and select the 'Submissions Needing Revision' folder to locate your manuscript file.

Please include the following items when submitting your revised manuscript:

- A rebuttal letter that responds to each point raised by the editor and reviewer(s). You should upload this letter as a separate file labeled 'Response to Reviewers'.
- A marked-up copy of your manuscript that highlights changes made to the original version. You should upload this as a separate file labeled 'Revised Manuscript with Track Changes'.
- An unmarked version of your revised paper without tracked changes. You should upload this as a separate file labeled 'Manuscript'.

Guidelines for resubmitting your figure files are available below the reviewer comments at the end of this letter.

We look forward to receiving your revised manuscript.

Kind regards,

Katherine Demi Kokkinias, Ph.D.

Staff Editor

PLOS Global Public Health

Journal Requirements:

1. Please include a complete copy of PLOS’ questionnaire on inclusivity in global research in your revised manuscript. Our policy for research in this area aims to improve transparency in the reporting of research performed outside of researchers’ own country or community. The policy applies to researchers who have travelled to a different country to conduct research, research with Indigenous populations or their lands, and research on cultural artefacts. The questionnaire can also be requested at the journal’s discretion for any other submissions, even if these conditions are not met.  Please find more information on the policy and a link to download a blank copy of the questionnaire here: https://journals.plos.org/globalpublichealth/s/best-practices-in-research-reporting. Please upload a completed version of your questionnaire as Supporting Information when you resubmit your manuscript.

2. Please amend your detailed Financial Disclosure statement. This is published with the article. It must therefore be completed in full sentences and contain the exact wording you wish to be published.

i. State the initials, alongside each funding source, of each author to receive each grant. For example: "This work was supported by the National Institutes of Health (####### to AM; ###### to CJ) and the National Science Foundation (###### to AM)."

3. Please ensure that your Ethics Statement is available in its entirety at the beginning of your Methods section, under a subheading 'Ethics Statement'.

4. Please provide separate figure files in .tif or .eps format.

For more information about figure files please see our guidelines:

https://journals.plos.org/globalpublichealth/s/figures

https://journals.plos.org/globalpublichealth/s/figures#loc-file-requirements

5. Please upload separate figure files in .tif or .eps format. Also, remove the figures from your manuscript file but keep the legends.

6. In the online submission form, you indicated that “De-identified survey datasets supporting the quantitative analysis are available from the corresponding author upon reasonable request, in accordance with IRB restrictions and ethical approval.”.

All PLOS journals now require all data underlying the findings described in their manuscript to be freely available to other researchers, either

1. In a public repository,

2. Within the manuscript itself, or

3. Uploaded as supplementary information.

This policy applies to all data except where public deposition would breach compliance with the protocol approved by your research ethics board. If your data cannot be made publicly available for ethical or legal reasons (e.g., public availability would compromise patient privacy), please explain your reasons by return email and your exemption request will be escalated to the editor for approval. Your exemption request will be handled independently and will not hold up the peer review process, but will need to be resolved should your manuscript be accepted for publication. One of the Editorial team will then be in touch if there are any issues.

If the reviewer comments include a recommendation to cite specific previously published works, please review and evaluate these publications to determine whether they are relevant and should be cited. There is no requirement to cite these works unless the editor has indicated otherwise.

Additional Editor Comments (if provided):

[Note: HTML markup is below. Please do not edit.]

Reviewers' comments:

Reviewer's Responses to Questions

**Comments to the Author**

1. Does this manuscript meet PLOS Global Public Health’s publication criteria?

Reviewer #1: Yes

**********

2. Has the statistical analysis been performed appropriately and rigorously?

Reviewer #1: No

**********

3. Have the authors made all data underlying the findings in their manuscript fully available (please refer to the Data Availability Statement at the start of the manuscript PDF file)?

The PLOS Data policy

Reviewer #1: Yes

**********

4. Is the manuscript presented in an intelligible fashion and written in standard English?

Reviewer #1: Yes

**********

Reviewer #1: The manuscript investigates stakeholder perspectives on establishing student-run free clinics (SRFCs) to strengthen non-communicable disease (NCD) care in Ghana. The study employs an exploratory sequential mixed-methods design, starting with qualitative focus groups and interviews with students, faculty, Ghana Health Service officials, and community members, followed by a quantitative survey of 316 students. Findings indicate strong support for SRFCs as feasible, contextually appropriate, and complementary to existing primary care initiatives.

The topic is timely and relevant as Ghana faces a rising NCD burden amid shortages in the healthcare workforce. The paper contributes original empirical data from a low-resource context where SRFCs remain underexplored. The manuscript is clear, well-organized, and adheres to mixed-methods conventions, though certain areas—particularly integration depth and analytic justification—require tightening before publication.

Strengths

1. Addresses an under-researched intervention in sub-Saharan Africa.

2. Well-structured mixed-methods design with clear sequential logic.

3. Comprehensive stakeholder representation (students, community, institutional leaders).

4. Strong applied implications for health systems and education policy.

Weaknesses

1. Lack of analytical rigor in qualitative analysis

- Purposive sampling in the qualitative phase is appropriate, but inclusion criteria for key informants and community members need clearer justification.

- Explain how saturation was determined.

- Thematic analysis appears descriptive rather than interpretive.

- Member-checking and coder triangulation are mentioned but not evidenced. Include a summary table or description of inter-coder reliability (e.g., Cohen’s κ).

2. Quantitative analysis requires more rigorous reporting of procedures and results.

- Clarify whether the survey items derived from qualitative themes underwent pilot validation, and include evidence of reliability (e.g., Cronbach's alpha, content and face validity, etc).

- The sample of 316 students is sizable, but the sample size calculation is not reported. Authors should specify assumptions and formula used to derive this number (expected effect size, power, confidence level).

- The logistic regression results (OR = 1.41, p = 0.007) are presented without model fit indices, confidence intervals, or assumption testing. Include 95% CIs, and specify checks for multicollinearity and goodness-of-fit (e.g., Hosmer–Lemeshow test).

- State whether the Chi-square assumptions (expected frequencies ≥ 5, independence of observations) were verified, given the relatively small subgroup counts. A re-analysis using Fisher’s Exact Test is warranted if those assumptions were not met.

- Clarify on what was being tested using the Chi-square test: association (as stated in line 309) or differences (as stated in line 198)?

- A list of operational definitions of quantitative variables should be included.

3. The discussion overstates generalizability and lacks a critical reflection on implementation challenges.

- The discussion should address structural barriers such as regulatory oversight, sustainable funding, and faculty load. These need to be supported by citations.

4. Figures and tables are numerous, but some could be synthesized or relegated to supplementary materials.

-Some figures (e.g., Figures 3–6) depict near-identical rating scales. Consider combining or summarizing to improve readability.

-Tables 3–5 contain redundant information; Table numbering should be corrected (two Table 3s appear).

Overall Recommendation: Major revision (methodological clarification and analytic refinement required before acceptance).

**********

what does this mean?). If published, this will include your full peer review and any attached files.

**Do you want your identity to be public for this peer review?**  If you choose “no”, your identity will remain anonymous but your review may still be made public.

For information about this choice, including consent withdrawal, please see our Privacy Policy

Reviewer #1: **Yes:** Mohd Nazan, Ahmad Iqmer Nashriq

**********

[NOTE: If reviewer comments were submitted as an attachment file, they will be attached to this email and accessible via the submission site. Please log into your account, locate the manuscript record, and check for the action link "View Attachments". If this link does not appear, there are no attachment files.]

# Author comment: Author response to Decision Letter 1

**DOI:** 10.1371/journal.pgph.0005092.r002

29 Nov 2025

Attachment Submitted filename: Response to the Reviewers.docx

# Peer review: Decision Letter 1

**DOI:** 10.1371/journal.pgph.0005092.r003

**Authors:** Jen Edwards

25 Jan 2026

PGPH-D-25-02203R1

Exploring Stakeholder Support for Student-Run Free Clinics to Address Gaps in Non-Communicable Disease Care in Ghana: An Exploratory Sequential Mixed-Methods Study.

PLOS Global Public Health

Dear Dr. Fleischer,

Thank you for submitting your manuscript to PLOS Global Public Health. After careful consideration, we feel that it has merit but does not fully meet PLOS Global Public Health’s publication criteria as it currently stands. Therefore, we invite you to submit a revised version of the manuscript that addresses the points raised during the review process.

Please submit your revised manuscript by Feb 22 2026 11:59PM. If you will need more time than this to complete your revisions, please reply to this message or contact the journal office at globalpubhealth@plos.org. When you're ready to submit your revision, log on to https://www.editorialmanager.com/pgph/ and select the 'Submissions Needing Revision' folder to locate your manuscript file.

Please include the following items when submitting your revised manuscript:

- A letter that responds to each point raised by the editor and reviewer(s). You should upload this letter as a separate file labeled 'Response to Reviewers'.
- A marked-up copy of your manuscript that highlights changes made to the original version. You should upload this as a separate file labeled 'Revised Manuscript with Track Changes'.
- An unmarked version of your revised paper without tracked changes. You should upload this as a separate file labeled 'Manuscript'.

Guidelines for resubmitting your figure files are available below the reviewer comments at the end of this letter.

We look forward to receiving your revised manuscript.

Kind regards,

Jen Edwards

Staff Editor

PLOS Global Public Health

Journal Requirements:

1. Please include a complete copy of PLOS’ questionnaire on inclusivity in global research in your revised manuscript. Our policy for research in this area aims to improve transparency in the reporting of research performed outside of researchers’ own country or community. The policy applies to researchers who have travelled to a different country to conduct research, research with Indigenous populations or their lands, and research on cultural artefacts. The questionnaire can also be requested at the journal’s discretion for any other submissions, even if these conditions are not met.  Please find more information on the policy and a link to download a blank copy of the questionnaire here: https://journals.plos.org/globalpublichealth/s/best-practices-in-research-reporting. Please upload a completed version of your questionnaire as Supporting Information when you resubmit your manuscript.

If the reviewer comments include a recommendation to cite specific previously published works, please review and evaluate these publications to determine whether they are relevant and should be cited. There is no requirement to cite these works unless the editor has indicated otherwise.

Please review your reference list to ensure that it is complete and correct. If you have cited papers that have been retracted, please include the rationale for doing so in the manuscript text, or remove these references and replace them with relevant current references. Any changes to the reference list should be mentioned in the rebuttal letter that accompanies your revised manuscript. If you need to cite a retracted article, indicate the article’s retracted status in the References list and also include a citation and full reference for the retraction notice.

Additional Editor Comments (if provided):

[Note: HTML markup is below. Please do not edit.]

Reviewers' comments:

Reviewer's Responses to Questions

**Comments to the Author**

Reviewer #2: All comments have been addressed

**********

publication criteria?

Reviewer #2: Yes

**********

3. Has the statistical analysis been performed appropriately and rigorously?

Reviewer #2: Yes

**********

4. Have the authors made all data underlying the findings in their manuscript fully available (please refer to the Data Availability Statement at the start of the manuscript PDF file)?

The PLOS Data policy

Reviewer #2: Yes

**********

5. Is the manuscript presented in an intelligible fashion and written in standard English?

Reviewer #2: Yes

**********

Reviewer #2: The quantitative survey heavily sampled students from public universities, with very limited representation from private institutions. This limits the generalizability of findings on student support for SRFCs. The manuscript would benefit from its mention in limitation section.

**********

what does this mean?). If published, this will include your full peer review and any attached files.

**Do you want your identity to be public for this peer review?**  If you choose “no”, your identity will remain anonymous but your review may still be made public.

For information about this choice, including consent withdrawal, please see our Privacy Policy

Reviewer #2: **Yes:** Arohi Chauhan

**********

[NOTE: If reviewer comments were submitted as an attachment file, they will be attached to this email and accessible via the submission site. Please log into your account, locate the manuscript record, and check for the action link "View Attachments". If this link does not appear, there are no attachment files.]

# Author comment: Author response to Decision Letter 2

**DOI:** 10.1371/journal.pgph.0005092.r004

5 Feb 2026

Attachment Submitted filename: Response to Reviewers.docx

# Peer review: Decision Letter 2

**DOI:** 10.1371/journal.pgph.0005092.r005

**Authors:** Joel Francis

19 May 2026

PGPH-D-25-02203R2

Exploring Stakeholder Support for Student-Run Free Clinics to Address Gaps in Non-Communicable Disease Care in Ghana: An Exploratory Sequential Mixed-Methods Study.

PLOS Global Public Health

Dear Dr. Fleischer,

Thank you for submitting your manuscript to PLOS Global Public Health. After careful consideration, we feel that it has merit but does not fully meet PLOS Global Public Health’s publication criteria as it currently stands. Therefore, we invite you to submit a revised version of the manuscript that addresses the points raised during the review process.

Please submit your revised manuscript by Jun 18 2026 11:59PM. If you will need more time than this to complete your revisions, please reply to this message or contact the journal office at globalpubhealth@plos.org. When you're ready to submit your revision, log on to https://www.editorialmanager.com/pgph/ and select the 'Submissions Needing Revision' folder to locate your manuscript file.

Please include the following items when submitting your revised manuscript:

- A letter that responds to each point raised by the editor and reviewer(s). You should upload this letter as a separate file labeled 'Response to Reviewers'.
- A marked-up copy of your manuscript that highlights changes made to the original version. You should upload this as a separate file labeled 'Revised Manuscript with Track Changes'.
- An unmarked version of your revised paper without tracked changes. You should upload this as a separate file labeled 'Manuscript'.

Guidelines for resubmitting your figure files are available below the reviewer comments at the end of this letter.

As the corresponding author, your ORCID iD is verified in the submission system and will appear in the published article. PLOS supports the use of ORCID, and we encourage all coauthors to register for an ORCID iD and use it as well. Please encourage your coauthors to verify their ORCID iD within the submission system before final acceptance, as unverified ORCID iDs will not appear in the published article. *Only*  the individual author can complete the verification step; PLOS staff *cannot*  verify ORCID iDs on behalf of authors.

We look forward to receiving your revised manuscript.

Kind regards,

Joel Msafiri Francis, MD, MS, PhD

Academic Editor

PLOS Global Public Health

Journal Requirements:

If the reviewer comments include a recommendation to cite specific previously published works, please review and evaluate these publications to determine whether they are relevant and should be cited. There is no requirement to cite these works unless the editor has indicated otherwise.

Please review your reference list to ensure that it is complete and correct. If you have cited papers that have been retracted, please include the rationale for doing so in the manuscript text, or remove these references and replace them with relevant current references. Any changes to the reference list should be mentioned in the rebuttal letter that accompanies your revised manuscript. If you need to cite a retracted article, indicate the article’s retracted status in the References list and also include a citation and full reference for the retraction notice.

Additional Editor Comments (if provided):

[Note: HTML markup is below. Please do not edit.]

Reviewers' comments:

Reviewer's Responses to Questions

**Comments to the Author**

Reviewer #3: (No Response)

**********

publication criteria?

Reviewer #3: Yes

**********

3. Has the statistical analysis been performed appropriately and rigorously?

Reviewer #3: N/A

**********

4. Have the authors made all data underlying the findings in their manuscript fully available (please refer to the Data Availability Statement at the start of the manuscript PDF file)?

The PLOS Data policy

Reviewer #3: Yes

**********

5. Is the manuscript presented in an intelligible fashion and written in standard English?

Reviewer #3: Yes

**********

Reviewer #3: (No Response)

**********

what does this mean?). If published, this will include your full peer review and any attached files.

**Do you want your identity to be public for this peer review?**  If you choose “no”, your identity will remain anonymous but your review may still be made public.

For information about this choice, including consent withdrawal, please see our Privacy Policy

Reviewer #3: **Yes:** Elamin Mohamed

**********

[NOTE: If reviewer comments were submitted as an attachment file, they will be attached to this email and accessible via the submission site. Please log into your account, locate the manuscript record, and check for the action link "View Attachments". If this link does not appear, there are no attachment files.]

Attachment Submitted filename: Student-run free clinic study review.docx

# Author comment: Author response to Decision Letter 3

**DOI:** 10.1371/journal.pgph.0005092.r006

12 Jun 2026

Attachment Submitted filename: Authors Response to the Reviewers.docx

# Peer review: Decision Letter 3

**DOI:** 10.1371/journal.pgph.0005092.r007

**Authors:** Julia Robinson

3 Sep 2026

Exploring Stakeholder Support for Student-Run Free Clinics to Address Gaps in Non-Communicable Disease Care in Ghana: An Exploratory Sequential Mixed-Methods Study.

PGPH-D-25-02203R3

Dear Dr. Fleischer,

We are pleased to inform you that your manuscript 'Exploring Stakeholder Support for Student-Run Free Clinics to Address Gaps in Non-Communicable Disease Care in Ghana: An Exploratory Sequential Mixed-Methods Study.' has been provisionally accepted for publication in PLOS Global Public Health.

Before your manuscript can be formally accepted you will need to complete some formatting changes, which you will receive in a follow up email. A member of our team will be in touch with a set of requests.

Please note that your manuscript will not be scheduled for publication until you have made the required changes, so a swift response is appreciated.

IMPORTANT: The editorial review process is now complete. PLOS will only permit corrections to spelling, formatting or significant scientific errors from this point onwards. Requests for major changes, or any which affect the scientific understanding of your work, will cause delays to the publication date of your manuscript.

If your institution or institutions have a press office, please notify them about your upcoming paper to help maximize its impact. If they'll be preparing press materials, please inform our press team as soon as possible -- no later than 48 hours after receiving the formal acceptance. Your manuscript will remain under strict press embargo until 2 pm Eastern Time on the date of publication. For more information, please contact globalpubhealth@plos.org.

Thank you again for supporting Open Access publishing; we are looking forward to publishing your work in PLOS Global Public Health.

Best regards,

Julia Robinson

Executive Editor

PLOS Global Public Health

***********************************************************

Reviewer Comments (if any, and for reference):

Reviewer's Responses to Questions

**Comments to the Author**

Reviewer #3: All comments have been addressed

**********

publication criteria?

Reviewer #3: Yes

**********

3. Has the statistical analysis been performed appropriately and rigorously?

Reviewer #3: N/A

**********

4. Have the authors made all data underlying the findings in their manuscript fully available (please refer to the Data Availability Statement at the start of the manuscript PDF file)?

The PLOS Data policy

Reviewer #3: Yes

**********

5. Is the manuscript presented in an intelligible fashion and written in standard English?

Reviewer #3: Yes

**********

Reviewer #3: I am satisfied with the authors' response to the comments raised.

I think it is a good study especially within our African context as the Student-run Free Clinic concept addresses access to care from the population prospectives and improves the quality of undergraduate education if managed appropriately.

I thank the authors for their response and the editorial team for the review invitation.

Dr. Elamin Mohamed

MBBS, M.FAM.MED, MPH, FCFP (SA), (MBA)*.

**********

what does this mean?). If published, this will include your full peer review and any attached files.

**Do you want your identity to be public for this peer review?**  If you choose “no”, your identity will remain anonymous but your review may still be made public.

For information about this choice, including consent withdrawal, please see our Privacy Policy

Reviewer #3: **Yes:** Dr. Elamin Mohamed

**********
